# Information maximization explains state-dependent synaptic plasticity and memory reorganization during non-rapid eye movement sleep

**DOI:** 10.1093/pnasnexus/pgac286

**Published:** 2022-12-10

**Authors:** Kensuke Yoshida, Taro Toyoizumi

**Affiliations:** Laboratory for Neural Computation and Adaptation, RIKEN Center for Brain Science, 2-1 Hirosawa, Wako, Saitama 351-0198, Japan; Department of Mathematical Informatics, Graduate School of Information Science and Technology, The University of Tokyo, 7-3-1 Hongo, Bunkyo-ku, Tokyo 113-8656, Japan; Laboratory for Neural Computation and Adaptation, RIKEN Center for Brain Science, 2-1 Hirosawa, Wako, Saitama 351-0198, Japan; Department of Mathematical Informatics, Graduate School of Information Science and Technology, The University of Tokyo, 7-3-1 Hongo, Bunkyo-ku, Tokyo 113-8656, Japan

**Keywords:** spike-timing-dependent plasticity, slow wave, efficient coding hypothesis, normative model, learning rule

## Abstract

Slow waves during the non-rapid eye movement (NREM) sleep reflect the alternating up and down states of cortical neurons; global and local slow waves promote memory consolidation and forgetting, respectively. Furthermore, distinct spike-timing-dependent plasticity (STDP) operates in these up and down states. The contribution of different plasticity rules to neural information coding and memory reorganization remains unknown. Here, we show that optimal synaptic plasticity for information maximization in a cortical neuron model provides a unified explanation for these phenomena. The model indicates that the optimal synaptic plasticity is biased toward depression as the baseline firing rate increases. This property explains the distinct STDP observed in the up and down states. Furthermore, it explains how global and local slow waves predominantly potentiate and depress synapses, respectively, if the background firing rate of excitatory neurons declines with the spatial scale of waves as the model predicts. The model provides a unifying account of the role of NREM sleep, bridging neural information coding, synaptic plasticity, and memory reorganization.

Significance Statement Some memories are consolidated, and others are forgotten during non-rapid eye movement sleep. This reorganization process is considered to involve synaptic plasticity in the presence of slow waves characterized by alternating up and down states at distinct spatial scales. Previous theories do not consider such patterns of slow waves and give only partial explanations of the reorganization process. Here, we report that the information maximization principle provides a unifying account for two essential experimental findings: the distinct synaptic plasticity during up and down states in slow waves and the opposite memory reorganization effects during global and local slow waves. The theory bridges neural coding, synaptic plasticity, and memory reorganization in non-rapid eye movement sleep.

Sleep is an essential physiological process and is widely conserved across species. One proposed role of sleep is to reorganize memory by regulating synaptic plasticity; some memories of awake experiences are consolidated, whereas others are forgotten (1–3). Multiple studies have explored the mechanism behind memory consolidation and forgetting by focusing on slow waves observed during non-rapid eye movement (NREM) sleep. Slow waves are low-frequency (<4 Hz) waves in electroencephalography (EEG) and local field potential (LFP), and their presence distinguishes NREM sleep from awake or rapid eye movement (REM) sleep. Each cortical neuron shows low-frequency transitions between the up (depolarized membrane potential) and down (hyperpolarized membrane potential) states synchronously to slow waves (4). Both the correlational and causal relationships between slow waves and memory consolidation have been established. It has been reported that slow-wave activity is correlated with task performance after sleep (5), and boosting slow waves can enhance memory consolidation (6–9).

Several studies have suggested that slow waves should be separated into distinct classes (10–16). Although different classification schemes have been used in previous studies, one of the classes is more global, while the other is more local (10, 12–14). A recent study further suggested that these two classes of slow waves have opposite effects on memory reorganization; the global and local classes promote memory consolidation and forgetting, respectively (13). These studies suggest that memory reorganization is induced depending on the subtle sleep states, such as the up and down states of global and local slow waves.

One possible explanation for how these sleep states differentially modulate the memory reorganization is that synaptic plasticity is modulated depending on the sleep state. Previous studies have shown that neuronal activity patterns in the awake state are reactivated within the slow waves during NREM sleep (17–19). Although the synaptic plasticity rule during NREM sleep is largely unknown, a recent experimental study using anesthetized young mice in vivo has suggested that the spike-timing-dependent plasticity (STDP) during up states is biased toward depression compared with down states (20). Consistently, another experimental study using acute brain slices demonstrated that the subthreshold inputs during up but not down states induce synaptic weakening (21). These findings suggest that neuronal reactivation can induce different synaptic plasticity in the up and down states. This difference might be the key to understanding memory reorganization during NREM sleep and raises two further issues worth exploring theoretically. First, what is the benefit of modulating the synaptic plasticity rule depending on the up and down states? Because the nervous system has evolved to work efficiently, the efficiency of neuronal coding might be enhanced by this modulation. Second, how does the state-dependent synaptic plasticity reorganize memories in global and local slow waves?

To understand these issues, we adopted a normative approach based on the information maximization (infomax) principle (22, 23) and derived a synaptic plasticity rule for a spiking neuron model (24, 25) that achieves efficient information transmission. We found that the baseline firing rate is an important parameter of the infomax rule. An increased baseline firing rate biases the synaptic plasticity towards depression, consistent with the reported difference in STDP between the up and down states. We then constructed a neuronal network model exhibiting global and local slow waves and showed that four states (up and down states of global and local slow waves) have distinct STDP owing to different baseline firing rates. Finally, we suggest that the difference in synaptic plasticity in global and local slow waves can set a balance between memory consolidation and forgetting, consistent with the previous experimental findings (see Fig. 6 for a schematic summary).

## Results

### Optimal synaptic plasticity is biased toward depression in high firing rates

To consider optimal synaptic plasticity in different sleep states, we first considered a feedforward network model with a postsynaptic neuron and multiple excitatory presynaptic neurons, which we call the single-neuron model. In this model, presynaptic spikes at synapse *j* evoked excitatory postsynaptic potentials (EPSPs) with the amplitude *w_j_* and exponential decays with a time constant of 25 ms. The membrane potential of the postsynaptic neuron was computed as *u*(*t*) = *u_r_* + ∑_*j*_*w_j_h_j_*(*t*), where *u_r_* is the resting membrane potential and *h_j_* is the EPSP time-course from presynaptic neuron *j* with an instantaneous increment of 1 after each presynaptic spike. The postsynaptic neuron emits spikes with firing probability density *g*^E^(*u*(*t*))*R*(*t*), where *g*^E^(*u*) is a softplus activation intensity function, and refractory factor *R*(*t*) models the transient suppression of the postsynaptic firing rate after a postsynaptic spike (see the “Methods” section). Following the infomax approach, we derive the optimal synaptic plasticity rule for maximizing information transmission while synaptic weights are constrained by their cost. We assumed that the synaptic weights *w_j_* change following the gradient of the utility function, which is the mutual information between the presynaptic and postsynaptic spikes minus the synaptic weight cost (24, 25). Thus, the synaptic weight changes were described by $\frac{dw_j}{dt} \propto \frac{dI}{dw_j} - \lambda \frac{d\Phi }{dw_j}$ with mutual information *I* and synaptic weight cost Φ (see the “Methods” section). The cost term Φ includes the square of the synaptic weight, which eliminates the synaptic weights that do not contribute to information transmission. Coefficient λ controls the importance of the synaptic cost term relative to the information term. We omitted the homeostatic term assumed in the previous studies because it did not contribute to our results, where the postsynaptic firing rate was kept on average within the homeostatic range. The gradient of mutual information *dI*/*dw_j_* was explicitly derived and can be computed in real-time using only the variables observable at synapse *j*, namely, the EPSP time-course *h_j_*, postsynaptic spikes informed by a back-propagating action potential, postsynaptic activation intensity *g*^E^(*u*) as a function of postsynaptic membrane potential *u*, refractory factor *R*, and mean activation intensity ${\bar{g}}$ (see the “Methods” section). In addition, the gradient of the cost term decreased the synaptic strength by λ*w_j_* for every presynaptic spike of neuron *j*.

**Fig. 1.**
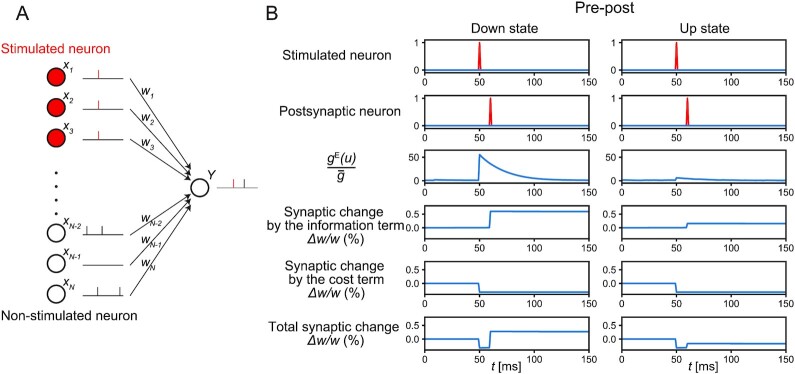
The infomax rule in the single-neuron model. (A) A single-neuron model of synaptic plasticity. Stimulated neurons synchronously emit spikes upon external presynaptic stimulation, and their synaptic weights change according to the infomax rule, whereas nonstimulated neurons spontaneously emit Poisson spikes and their synaptic weights are fixed for simplicity. A postsynaptic neuron emits both spontaneous spikes and evoked spikes upon external postsynaptic stimulation. The red vertical bars represent evoked spikes. (B) Representative traces of a stimulated neuron’s activity, the postsynaptic neuron’s activity, the ratio of the momentary and mean activation intensity $g^{\mathrm{E}}(u)/\bar{g}$, and changes of a synaptic weight from a stimulated neuron in the down and up states. Synaptic changes by the infomax rule were computed by summing the effects of the information term $\frac{dI}{dw_j}$ and cost term $\frac{d\Phi }{dw_j}$. The synaptic increase by the information term was smaller in the up state than that in the down state.

We ran simulations imitating the experimental STDP protocols in vivo (20) in the single-neuron model. To mimic the experimental setup, we divided the presynaptic neurons into the stimulated and nonstimulated neurons (Fig. 1A). Twenty stimulated neurons synchronously emitted a spike upon external presynaptic stimulation, and their synaptic weights changed according to the infomax rule. One hundred nonstimulated neurons spontaneously emitted Poisson spikes at 2.0 and 0.1 Hz in the up and down states, respectively, while their synaptic weights were fixed for simplicity. The mean postsynaptic activation intensity, ${\bar{g}}$, was computed by taking the average of *g*^E^(*u*) in each state. This yielded ${\bar{g}}^{(u)} \approx 5.9$ Hz and ${\bar{g}}^{(d)} \approx 0.5$ Hz for the up and down states, respectively.

We first characterized synaptic changes induced by pre-post stimulation, where a presynaptic spike was induced 10 ms before the postsynaptic spike. Representative traces of a synaptic weight from a stimulated neuron in the up and down states are plotted in Fig.   1(B). The increase in $g^{\mathrm{E}}(u)/{\bar{g}}$ after presynaptic stimulation was greater when the mean firing rate was low, indicating that a postsynaptic spike can transmit a greater amount of information at a lower mean firing rate. Consequently, the information term caused a greater synaptic potentiation in the down state than that in the up state. The amount of synaptic potentiation due to the information term was roughly proportional to $\log (1+\Delta g/{\bar{g}})/({\bar{g}}+\Delta g)$, where $\Delta g = g^{\mathrm{E}}(u)-{\bar{g}}$ represents the increment in activation intensity due to the presynaptic stimulation (see the “Methods” section for details). Intuitively, Δ*g* measured the reliability of a synapse for transmitting the signal, and ${\bar{g}}$ represented the noise level that quantifies the frequency of the postsynaptic spikes in the background. Thus, $\Delta g/{\bar{g}}$ corresponds to the signal-to-noise ratio. By contrast, the change in the synaptic weight by the cost term was −λ*w_j_* after every presynaptic spike of neuron *j*, regardless of the mean firing rate.

**Fig. 2.**
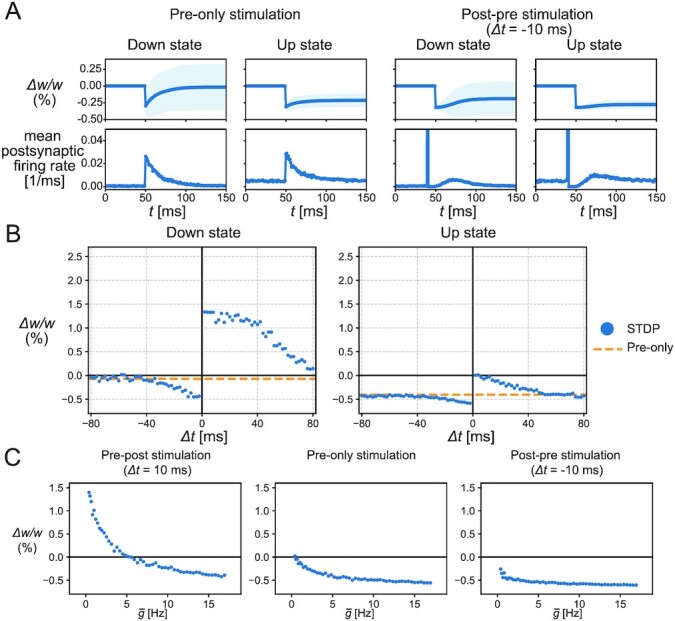
The synaptic plasticity induced by the STDP stimulations. (A) The mean traces of a synaptic weight and the mean postsynaptic firing rate in the pre-only stimulations or the post-pre stimulations with Δ*t* = −10 ms, where the stimulated neurons emitted a synchronous spike upon the external stimulation at *t* = 50 ms. In the post-pre stimulations, the postsynaptic neuron emitted an evoked spike at *t* = 40 ms and also responded to the presynaptic stimulation at *t* = 50 ms with some delay due to refractoriness. After the pre-only stimulations, the synaptic weight changed little in the down state, but was depressed in the up state. In the post-pre stimulation, the synaptic weight was depressed in both the down and up states. The lines and shadows of the weight traces represent the means and SDs, respectively. (B) The synaptic changes by the STDP stimulations (blue points) and pre-only stimulations (orange dotted lines). As the value of Δ*t* increased or decreased, the synaptic changes by the STDP stimulations converged to the change by the pre-only stimulations. Synaptic plasticity was biased towards depression in the up state. (C) Synaptic changes were dependent on mean activation intensity. The synaptic changes in pre-post stimulation with Δ*t* = 10 ms and pre-only stimulation decreased with increasing mean activation intensity, whereas the synaptic changes in post-pre stimulation with Δ*t* = −10 ms were less sensitive to mean activation intensity.

Fig. 1(B) displays the representative traces of synaptic weight; however, synaptic changes also depended on other postsynaptic spikes and presynaptic spikes from nonstimulated neurons, which can occur randomly. Below, we quantify the average synaptic changes induced by three kinds of stimulation: pre-only stimulation and post-pre stimulation (a presynaptic spike was induced 10 ms after an induced postsynaptic spike), in addition to the pre-post stimulation explored above. We started by simulating the pre-only stimulation. Experimentally, pre-only stimulation in the down state did not significantly change the synaptic weights of stimulated neurons (20). To reproduce this experimental result, we set the coefficient of the cost term to λ = 0.32 (mV)^−2^, so that the changes in the stimulated synapses were on average zero in the down state (Fig. 2A). This value of λ was used throughout this paper. Simulations of the up state showed an overall synaptic depression because the synaptic potentiation due to the information term decreased with the mean firing rate for the reason described above. In addition, neither artificial postsynaptic depolarization to the up state level in down states nor hyperpolarization to the down state level in up states appreciably affected the synaptic changes in the current setup (SI Appendix, Fig. S2), consistent with the experimental results (20).

Next, if a postsynaptic spike was evoked before the presynaptic stimulation (i.e., post-pre stimulation), the infomax rule caused the synaptic depression both in the up and down states because the induced postsynaptic spike before the presynaptic stimulation reduced the value of *R*(*t*) and prevented the synaptic weights from increasing by the information term, whereas the cost term could still decrease these synapses (Fig. 2A).

To investigate how the STDP window of the infomax rule differs in the up and down states, the time difference between presynaptic and postsynaptic stimulations was systematically changed in the single-neuron model. Consistent with the observations above, the entire STDP curve was biased toward synaptic depression in the high mean firing rate condition (Fig. 2B). While the synaptic change caused by post-pre stimulation was relatively insensitive to mean firing rates, the high mean firing rates biased the synaptic changes toward depression with the pre-post and pre-only stimulations (Fig.   2C). These results were consistent with the corresponding in-vivo experimental results (20). In addition, while the experimental results are limited to a few representative time differences (within 10 ms and within −10 ms in the down states and 10, 50, and −10 ms in the up states) (20), the infomax model predicted the whole STDP curve. Although this tendency of synaptic depression induced by the high mean firing rates did not qualitatively depend on the choices of the activation intensity function *g*^E^(*u*) except for the pure exponential function (see SI Appendix, Fig. S1 and the “Methods” section), the exact position of STDP curve and the activity threshold, separating potentiation and depression under the pre-only stimulations, depended on several parameters (SI Appendix, Figs. S3 and S4). Especially, the pre-only stimulation with small synaptic weights and a large number of the stimulated neurons tended to induce potentiation even in up states, albeit to a lesser degree than down states (SI Appendix, Fig. S4). In summary, the modulation of the infomax rule by the mean firing rate explained the synaptic plasticity during the up and down states in slow waves.

### Firing rates of excitatory neurons are higher during local slow waves than those during global slow waves

**Fig. 3.**
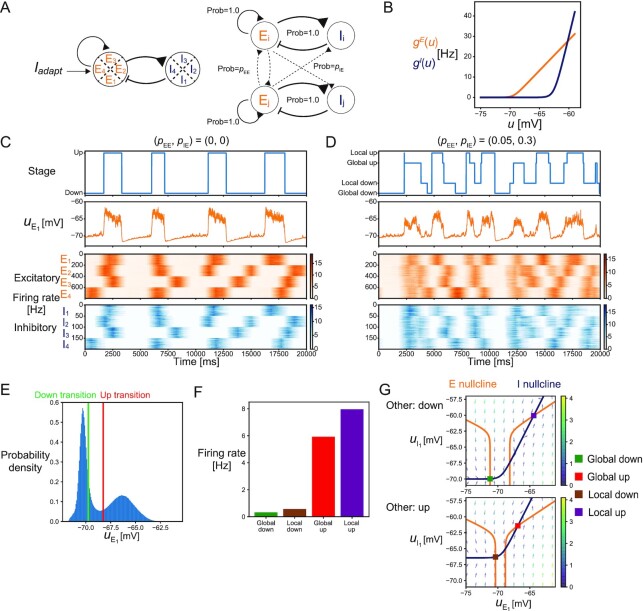
The cortical network model exhibiting global and local slow waves. (A) The schematic description of the model. (Left) Model composed of four local networks. Each local network included 200 excitatory and 50 inhibitory neurons. (Right) Within a network, connections existed between two excitatory neurons, and between excitatory and inhibitory neurons, while two inhibitory neurons had no connections between them. Between different networks, there were sparse connections from excitatory to excitatory or inhibitory neurons but none from the inhibitory neurons. (B) The activation functions of excitatory and inhibitory neurons. Both functions were softplus functions, but the threshold and slope were greater for inhibitory neurons than those for the excitatory neurons. (C) The dynamics of the slow-wave model in the case of no connections between different local networks. The up and down states of the E1 population were classified using the mean membrane potential $u_{\mathrm{E}_1}$. The four networks independently transited between the up and down states. (D) The dynamics of the slow-wave model in the case that there exist sparse connections between different networks. The up and down states of the E1 population were classified into the global or local states depending on the states of the other populations. (E) The probability density of the membrane potential. The transition thresholds to the up and down states are indicated by red and green lines, respectively. (F) The mean firing rates of the E1 population in global down, local down, global up, and local up states. (G) The phase plane of the E1 and I1 population in the case that other populations are in down states (upper) or in up states (lower) (see SI Appendix Methods for details). Excitatory and inhibitory nullclines are shown as orange and blue lines, respectively. The four steady points corresponding to global down, local down, global up, and local up states are shown; the up state of E1 population is global or local when the other populations are in the up or down states, respectively, while the down state of E1 population is global or local when the other populations are in the down or up states, respectively. The $u_{\mathrm{E}_1}$ values of four steady points followed global down < local down < global up < local up in ascending order.

To study how the above-mentioned findings would apply to memory reorganization during NREM sleep, we constructed the network models of cortical neurons that generate slow waves, which we call the slow-wave model. We started by constructing a spatially homogeneous model similar to that in ref. (26) and then introduced spatial heterogeneity to produce local and global slow waves. The slow-wave model consisted of recurrently connected spiking neurons, including the excitatory and inhibitory neurons (see the “Methods” section). The spatially homogeneous model assumed no connections between two inhibitory neurons but all-to-all connections between two excitatory neurons and between excitatory and inhibitory neurons. Each excitatory neuron had adaptation currents that accumulated with the spikes. Adaptation currents correspond to, for example, the potassium currents involved in generating slow waves (27–30). The activation functions of excitatory and inhibitory neurons were both modeled by softplus functions, but the threshold and slope were greater for the inhibitory neurons than those for the excitatory neurons (Fig. 3B). In these settings, the excitatory and inhibitory activities showed bistability of the up and down states, and the transitions were caused by the slowly changing adaptation currents (SI Appendix, Fig. S5A and B). This was consistent with the previous theoretical study (26). The inhibitory population was mostly inactive in down states because of the high threshold (Fig. 3B), but was active in the up state and stabilized the recurrent excitatory activity.

To consider the difference between global and local slow waves, we extended the model by embedding four local networks, each as described above, within the overall network (Fig. 3A). In cases of no between-network connections, each local network independently produced up and down cycles of slow waves (Fig. 3C). Next, we introduced sparse long-range excitatory connections between different local networks. We assumed that the long-range connections project to both the excitatory and inhibitory neurons, as demonstrated in previous modeling studies (31). In this setting, each local network showed the transitions between the up and down states, some of which were local, whereas others were global in synchrony across the local networks (Fig. 3D). To objectively define global and local slow waves, we first classified the up and down states of each local network based on the mean membrane potential averaged across excitatory neurons (Fig. 3C and E). Transitions to the down and up states were detected when the mean membrane potential decreased below −69.75 mV and exceeded −68.25 mV, respectively (the choice of these thresholds did not affect the results; see SI Appendix, Fig. S6). We classified each state into a global or local state by counting the number of up and down states across the local networks (Fig. 3D) (see the “Methods” section). In the global up/down states, either all or all but one local network simultaneously achieved the same state.

We then analyzed how the difference between global and local slow waves affected the learning by the infomax rule. Since the outcome of the infomax rule depends on the mean firing rates, we examined the mean firing rates in the up and down states during the global and local slow waves. The mean firing rates of excitatory neurons followed global down < local down < global up < local up in ascending order in the simulations (Fig.   3F). The difference between the global and local down states was simply explained by the strength of the long-range excitation from the surrounding networks to the local excitatory population. Because the surrounding excitatory populations had elevated activity in their up state, the long-range excitation was stronger in the local down states than that in the global down states. Note that the local inhibitory population was mostly inactive in both the local and global down states and did not contribute significantly to the difference. By contrast, the difference between the global and local up states was mainly explained by the local inhibition to the excitatory population. While the local network was in the up state, its inhibitory activity was more sensitive to the long-range excitation than its excitatory activity because of the steeper inhibitory activation function at high membrane potential (see Fig. 3B and the “Methods” section). Therefore, the strong long-range excitation from the surrounding networks in the global up state effectively reduced the local excitatory activity via local inhibition. To verify this, we performed a phase plane analysis, assuming a large number of neurons (see SI Appendix Methods). The phase planes showed that the firing rates of the two stable points (i.e., up and down states) changed depending on the state of the surrounding networks (Fig. 3G and see SI Appendix Methods for parameter dependency). As expected, the membrane potential of the excitatory neurons was higher in local down states with elevated long-range excitation than that in the global down states. In addition, the membrane potential of excitatory neurons was lower in the global up states than that in the local up states. In this case, the long-range excitation shifted both the excitatory and inhibitory nullclines. Since the shift of the inhibitory nullcline was much larger than that of the excitatory nullcline, the membrane potential of excitatory neurons decreased with the long-range excitation (Fig. 3G). The observed higher local excitatory activity in the local up states than that of the global up states is a natural consequence of unstable recurrent excitatory dynamics being stabilized by the strong inhibition. A similar response to external input was previously demonstrated both experimentally and theoretically as a property of inhibition-stabilized networks (ISNs) (31–33). Based on these results, we hypothesized that the infomax rule, which is sensitive to the baseline firing rate, would yield distinct learning outcomes in the global and local slow waves.

### Optimal synaptic plasticity in up and down states of global and local slow waves

**Fig. 4.**
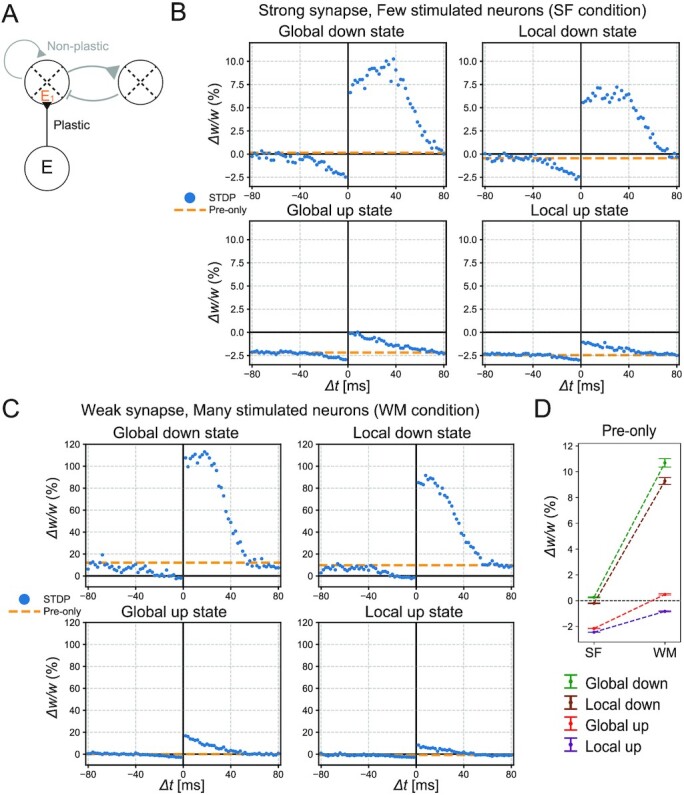
STDP in different sleep states. (A) The schematic description of the simulation. Presynaptic neurons had a feedforward projection onto an excitatory postsynaptic neuron in the E1 population. The feedforward synaptic weights were plastic, whereas the recurrent synaptic weights were fixed. (B) The synaptic changes caused by the STDP stimulations (blue points) and pre-only stimulations (orange dotted lines) during each sleep state in the case of strong synapses ($w_j^{\mathrm{ext}} = 0.5$ mV) and few stimulated neurons (*N*^ext^ = 20) (SF condition). As the value of Δ*t* increased or decreased, the synaptic changes by the STDP stimulations converged to the change by the pre-only stimulations. The amount of synaptic changes follows global down > local down > global up > local up, in descending order. (C) The synaptic changes caused by the STDP stimulations (blue points) and pre-only stimulations (orange dotted lines) during each sleep state in the case of weak synapses ( $w_j^{\mathrm{ext}} = 0.09$ mV) and many stimulated neurons (*N*^ext^ = 40) (WM condition). The synapses tend to be potentiated even in up states, while the amount of synaptic changes follows global down > local down > global up > local up, in descending order, as with (B). Note that the scale is different from (B). (D) The synaptic changes caused by the pre-only stimulations. Error bars represent SEM. The synaptic changes were dominated by synaptic potentiation in the WM condition. Especially in the WM condition, the pre-only stimulations during global and local up states induced synaptic potentiation and depression, respectively.

To study the outcome of the infomax rule in global and local slow waves, we first explored the STDP window using the slow-wave model in the previous section. We introduced multiple presynaptic excitatory neurons that spike synchronously when externally stimulated, as shown in Fig. 2. These presynaptic neurons projected onto a randomly selected postsynaptic neuron in the first excitatory population, E1, of the slow-wave model (Fig. 4A). We assumed that these feedforward synaptic weights were updated by the infomax rule, whereas the recurrent synaptic weights were fixed. The changes in the feedforward synaptic weights averaged over random realizations of the model are shown below.

We first studied the STDP by evoking a postsynaptic spike at a fixed time, before or after the presynaptic stimulation to 20 external input neurons. Here, we assume strong synapses with 0.5 mV EPSPs from these neurons. In addition to this evoked spike, the postsynaptic neuron could generate other spikes triggered by the network activity. In the slow-wave model, the mean activation intensity ${\bar{g}}$ was computed by averaging the activation intensity *g*^E^(*u_i_*) of neuron *i* for neurons included within the same excitatory population (see the “Discussion” section for possible biological implementations). As expected from Figs. 2 and 3, the STDP results depended on the sleep states of the slow-wave model (Fig. 4B). These results indicate two important points. First, the synaptic plasticity in the up states is biased toward synaptic depression as compared with the down states, which is consistent with the experimental findings (20). Second, synaptic plasticity in the local up and down states is biased toward synaptic depression as compared with the global up and down states, respectively. This property results from the model prediction that the mean firing rates are higher in the local up and down states than those in the corresponding global states. As expected from SI Appendix, Fig. S4, stimulating many weak synapses (40 synapses with 0.09 mV EPSPs) tended to induce more potentiation (Fig. 4C, note that the scale is different from Fig. 4B), although the difference between global and local up and down states described above still existed (Fig. 4D). Especially when many weak synapses were stimulated, as in Fig. 4C, the pre-only stimulation during global up states caused synaptic potentiation, and that during local up states caused synaptic depression (Fig. 4D).

**Fig. 5.**
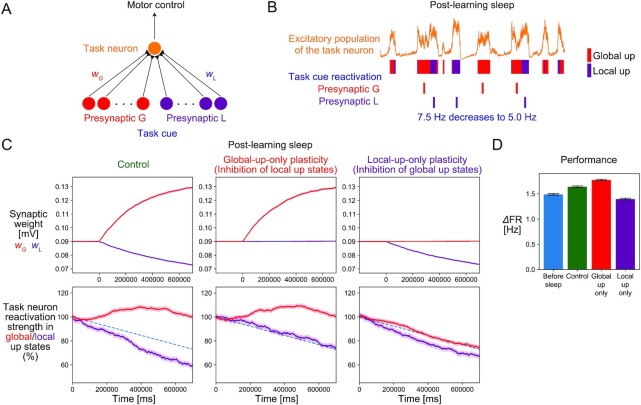
Changes in synaptic weights and task performance during post-learning NREM sleep. (A) The schematic description of the neuronal networks related to the task. Presynaptic neurons were divided into two populations *G* and *L*. Both *G* and *L* populations emitted synchronous spikes (“task cue”) during the task. The postsynaptic neuron (“task neuron”) is an excitatory neuron in the E1 population that is projected by the presynaptic neurons. Task performance is defined as the firing rate increase of the task neuron during the task period. (B) As neuronal reactivation, the *G* and *L* populations emitted synchronous spikes during the global and local up states of the E1 population during the post-learning NREM sleep, respectively, at the firing rates decreasing from 7.5 Hz at the beginning of sleep to 5.0 Hz at the end of sleep. (C) The changes of the synaptic weights and the task neuron reactivation strength during the post-learning sleep. The synaptic changes of the *G* and *L* populations are shown in red and purple, respectively (upper). The synapses of the population *G* were potentiated by reactivation during the global up states, whereas the synapses of the population *L* were depressed by reactivation during the local up states. When synaptic plasticity during the local-up or global-up is blocked, the synaptic changes of the corresponding population were inhibited. Hence, the sum of synaptic weights in two populations was global-up blocked < control < local-up blocked in ascending order. The reactivation strength of the task neuron in global and local up states are shown in red and purple, respectively (lower). The blue dotted line represents the assumed decrease in the task cue reactivation rate. The reactivation strengths of the task neuron in global and local up states were preserved and diminished, respectively, reflecting the synaptic changes of the corresponding population. In the local-up-blocked and global-up-blocked plasticity conditions, the reactivation strength of the task neuron in local and global up states became the same as the time-course of the task cue reactivation, respectively. The lines and shadows represent the means and SEMs in the 600 trials, respectively. (D) The comparison of task performance before and after synaptic changes during sleep. This tendency is the same as that of the sum of synaptic weights in the *G* and *L* populations shown in Fig. 5C. Error bars represent SEM.

To further investigate the impact of sleep states on memory reorganization, we simulate how synaptic weights that contribute to task performance change during subsequent sleep. This time, we simulated presynaptic neurons having small initial synaptic weights. The assumption is that relatively weak synapses are mainly involved in the in-vivo learning of a new task. During the awake condition, we assume that the presynaptic neurons emitted spikes synchronously upon the presentation of a task cue and projected to a postsynaptic neuron (task neuron) in the E1 population of the slow-wave model (Fig. 5A). The simulation was repeated over random realizations of the model parameters. Inspired by the brain–machine-interface task (13), we defined task performance as an increase in the task neuron’s firing rate upon the presentation of a task cue. Then, we considered the synaptic changes during post-learning NREM sleep, assuming that the feedforward synaptic weights have already been potentiated to elevate the postsynaptic firing rate during the task. Experimentally, the triple coupling of slow waves, spindles, and reactivation is considered crucial for memory consolidation (2, 18, 34, 35), in which spindles are considered to promote synaptic plasticity by facilitating dendritic activities (2, 35–38). Therefore, we assumed that synaptic plasticity is induced by the reactivation inputs from presynaptic neurons in the presence of spindles. Although we did not explicitly model spindles in this study, we assumed that spindles are nested in slow waves when memory reactivation occurs in up states of the task neuron (see the “Discussion” section for details). Presynaptic neurons were divided into two populations, the global-up-reactivated neurons *G* and local-up-reactivated neurons *L*, each consisting of 40 presynaptic neurons and synchronously emitted spikes with Poisson statistics during global up and local up states, respectively. We assumed that the Poisson rate of the memory reactivation (17, 19) of the task cue decreased from 7.5 Hz at the beginning of an NREM sleep to 5.0 Hz at the end of an NREM sleep to reproduce experimental results of task neuron reactivation (Fig. 5C, see below for detail). We updated the feedforward synaptic weights during post-learning NREM sleep according to the infomax rule when presynaptic reactivation happened. In Fig. 5, we restricted memory reactivation to occur during the up states of the local network only (Fig. 5B) because the intervention of the up states, not down states, mainly affected the performance of the brain–machine-interface task (13, 39). More generally, memory reactivation might also happen in down states depending on the experimental setup (40). This possibility was also investigated in SI Appendix, Fig. S7. Further simulation details are described in SI Appendix Methods.

The feedforward synaptic weights of the populations *G* and *L* were further potentiated and depressed in the simulation of post-learning NREM sleep, respectively (Fig. 5C), because global and local up states promote synaptic potentiation and depression, respectively, with weak and many stimulated synapses (Fig. 4D). Task performance increased during sleep, reflecting the increased sum of synaptic weights of the population *G* and *L*, consistent with the experimentally suggested memory consolidation during NREM sleep (Fig. 5D). The reactivation strength of the task neuron (i.e., the firing rate increase of the task neuron from baseline firing rates when the task cue is reactivated) during global and local up states were kept constant and decreased, respectively (Fig. 5C). Under the gradual decrease of the task cue reactivation rate, synaptic potentiation of the population *G* and depression of the population *L* promoted the reactivation strength of the task neuron during global and local up states to be kept constant and to be further decreased, respectively. This was consistent with the experimental result that reactivation strengths in the spindles nested in global and local up states were preserved and weakened, respectively (13).

Next, we investigated the roles of global and local slow waves in memory reorganization separately by inhibiting synaptic plasticity either during global or local up states. Note that in the simulation, synaptic plasticity within 50 mec after global or local up states was also inhibited to eliminate synaptic plasticity during the transition states that is sensitive to arbitrary model assumptions (see SI Appendix Methods for detail). The synaptic depression of the population *L* was inhibited when synaptic plasticity was blocked during local up states. Further, synaptic potentiation of the population *G* was inhibited when synaptic plasticity was blocked during global up states (Fig. 5C). As a result, the task performance showed a greater increase in the former case but a decrease in the latter case (Fig. 5D). The results are consistent with experimental findings that global and local slow waves contribute to memory consolidation and forgetting, respectively (13). The reactivation strength of the task neuron exhibited changes monotonically related to synaptic weights. The decrease of the reactivation strength of the task neuron during local up states became slower when synaptic plasticity was blocked during local up states. The reactivation strength of the task neuron during global up states decreased when synaptic plasticity was blocked during global up states. This was consistent with the experimental result that inhibition of global up states promoted a gradual decrease of reactivation strength in the spindles nested in global up states (13).

These results suggest that the balance of global and local slow waves with distinct information transfer capacities regulates the spectrum of memory consolidation and forgetting via the infomax synaptic plasticity rule.

## Discussion

**Fig. 6.**
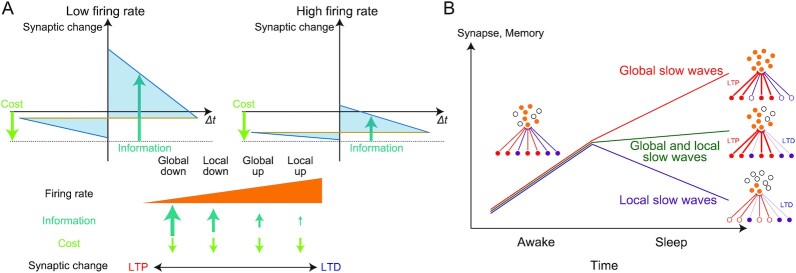
The proposed role of NREM sleep is to bridge neural information coding, synaptic plasticity, and memory reorganization. (A) The relationship between the mean firing rate and synaptic changes by the infomax rule. Synaptic potentiation by the information term was decreased at a high firing rate owing to many background spikes, whereas synaptic depression by the cost term was unaffected. Therefore, the high firing rate induced synaptic depression. Because the mean firing rates are global down < local down < global up < local up in ascending order, the amount of synaptic changes follows the opposite order. (B) The possible distinct roles of global and local slow waves. Reactivated patterns during global slow waves induced synaptic potentiation, whereas those during local slow waves induced synaptic depression. This could cause selective memory consolidation and forgetting of the reactivated patterns during the global and local slow waves, respectively.

Using a top-down approach with the information theory, we provided a unified learning rule, the infomax rule, for the state-dependent synaptic plasticity during NREM sleep. The infomax rule is comprised of the synaptic changes by the information term and synaptic depression by the synaptic cost term (Fig. 6A). A high firing rate condition biases the synaptic plasticity toward depression. The reason is that the signal-to-noise ratio for the synaptic transmission declines with the background firing rate of the postsynaptic neuron, and the cost term dominates the information term under a high firing rate condition. The learning rule yields an information-theoretical interpretation of different STDP observed in the up and down states (20). Moreover, it also provides the distinct STDP during the global and local slow waves, suggesting a possible mechanism for balancing memory consolidation and forgetting. These properties are consistent with the role of neuronal reactivation in global and local slow waves (13).

The infomax rule not only reproduces the biased STDP toward depression during up states (20), but also provides a reliable prediction of the entire STDP curve during up states; the original experiment measured the synaptic change using a few representative time differences (10, 50, and −10 ms) between the presynaptic and postsynaptic spikes. The infomax rule further predicts that the STDP curve is sensitive to the initial synaptic weights and number of synchronous inputs (SI Appendix, Figs. S3 and S4). These predictions are experimentally testable using protocols similar to those of ref. (20), using various time differences, and altering the strength of presynaptic stimulations. Importantly, the infomax rule also predicts that relatively weak synapses can be potentiated, even in the up state, if the surrounding area is also in the up state and if a large number of presynaptic neurons emit synchronous spikes (SI Appendix, Fig. S4). Consistent with the predicted synaptic potentiation of weak synapses during sleep, recent experimental findings suggest that synaptic potentiation and the formation of new spines play an essential role in memory consolidation (41–45). Therefore, the infomax rule provides a unifying view by reproducing both the state-dependent STDP (Fig. 4) and the wave-scale-dependent memory reorganization (Fig. 5). Note that the STDP curve during up states was measured in urethane-anesthetized young mice (20). Since the animal age or anesthesia could affect synaptic plasticity (46, 47), whether this STDP rule applies to physiological sleep in adult mice needs to be explored in future studies.

The infomax rule requires estimating the expected firing rate of each postsynaptic neuron in real-time to set the activity threshold separating the synaptic potentiation and depression. There are several biologically plausible implementations for computing this. The simplest estimate uses a temporally averaged firing rate. However, it tends to lag behind the true instantaneous firing rate in the presence of slow waves. This possibility is also at odds with the experimental observation, where manipulating postsynaptic membrane potential to be depolarized during down states or hyperpolarized during up states did not significantly affect the synaptic changes (20). This experimental result is reproduced if the expected instantaneous firing rate is accurately estimated using the average excitatory firing rate of the local network population because the artificial single-neuron manipulation does not significantly change the local population activity. It is possible that the inhibitory neurons projected by nearby excitatory neurons compute the average firing rate, which is consistent with the observation that inhibitory input could modulate the balance of synaptic potentiation and depression (48). Alternatively, astrocytes may temporally and spatially integrate nearby synaptic inputs and regulate the activity threshold separating synaptic potentiation and depression (49).

The infomax rule suggests the potential importance of down states for memory consolidation. Although some studies have assumed down states as resting periods for cellular maintenance (50), it has been reported that the hyperpolarization might be essential for slow waves to induce synaptic potentiation (44). A recent study further suggested that the neuronal activities during down states (“delta spikes”) coincided with the hippocampal ripple activities and might be important for memory consolidation (40). The infomax rule suggests that down states with fewer background spikes promote synaptic potentiation more than up states, implying that the delta spikes could effectively induce synaptic potentiation and memory consolidation. Many studies have also focused on the involvement of neuronal activities in up states or the transition period from down to up states during memory consolidation (13, 51–53). Such distinct roles of the neuronal reactivation during the up and down states warrant further study.

The infomax rule in our sleep model suggests the importance of the spatial scale of slow waves (12, 54). Specifically, the present model suggests that the synaptic changes induced by global slow waves are dominated by potentiation as compared to local slow waves because of different baseline firing rates. Although we did not explicitly model spindles in this study, including a spindle-generating mechanism to the model is an important future direction. In the current study, we simply assumed that slow-wave-nested spindles would be generated upon memory reactivation in the task neuron’s up states and they are necessary for inducing synaptic plasticity. This assumption agrees with the argument that the triple coupling of slow waves, spindles, and reactivation is crucial for memory consolidation (2, 18, 34, 35), in which spindles promote synaptic plasticity by enhancing dendritic activities (2, 35–38). Our model behavior is consistent under this assumption with the experimental results by Kim et al. Optogenetic inhibition of the primary motor cortex during global and local up states caused a decrease and increase in the ratio of the number of spindles nested in global slow waves to that in local slow waves, respectively (13). This result is expected if cortical activity in up states is needed to facilitate thalamic spindle generation (2, 15, 55). An extreme condition with the total lack of nested spindles during global or local up states corresponds to the lack of synaptic plasticity then (as explored in Fig. 5) according to the assumption. The suggested role of memory consolidation during global slow waves and forgetting during local slow waves raises the possibility that there are mechanisms regulating the spatial scales of slow waves for selecting a subset of reactivation events to be consolidated (Fig. 6B). One possibility is that some neuronal populations projecting broadly to cortical neurons promote global synchronization. Previous studies have consistently suggested that the thalamus (56) and claustrum (57) play a role in synchronizing the down states of multiple cortical neurons. If such neuronal populations are co-active with reactivation patterns, these patterns could be selectively consolidated. Considering the high temporal correlation between the hippocampal sharp-wave ripples (SWRs) and cortical slow waves (58), specific neuronal populations may regulate both the generation of slow waves and neuronal reactivation. This possibility needs to be explored further.

Our theory could be verified experimentally along with the following two points. First, excitatory firing rates during global up states were lower than during local up states. Although the direct evidence supporting this prediction has not been reported to our knowledge, it is indirectly supported by theoretical and experimental studies outside sleep research. Our network operates as an ISN in its up states (31–33, 59). ISN models reproduce the experimentally observed surround suppression effect, i.e., the reduction in both local excitatory and inhibitory firing rates upon the activation of surrounding populations (31, 33). This property is sufficient (but not necessary) for our model to exhibit lower excitatory firing rate in the up states of more global slow waves. Notably, a hallmark ISN property, the nonmonotonic inhibitory response, is experimentally verified across cortical areas (60). Furthermore, recent technical advances in neuronal recordings from a large number of neurons (61) can annotate up and down states in each local area more precisely. In the future, such recordings could directly verify the model prediction that the mean firing rates of excitatory neurons decline with the spatial scale of the slow waves. Another testable model prediction is that global slow waves should permit more efficient information transmission than local slow waves. For example, one could optogenetically stimulate a small group of neurons and quantify the accuracy of stimulus encoding in neurons postsynaptic to the stimulated neurons during global and local slow waves.

Another interesting perspective is the qualitative reorganization of memories during sleep (62). While our model focuses on synaptic plasticity and quantitative memory reorganization (i.e., consolidation vs. forgetting), a recent theory proposes that the learning cycle mimicking wakefulness, NREM sleep, and REM sleep promote the formulation of new cortical representations, not just strengthening or weakening experiences (63). Bridging synaptic plasticity rules mainly obtained in the rodent experiment and qualitative memory reorganization proposed in the cognitive study is an interesting future direction.

In summary, the proposed theory bridges neuronal information coding, synaptic plasticity, and memory reorganization. Our normative framework provides a versatile learning rule for state-dependent synaptic plasticity and memory reorganization during NREM sleep.

## Methods

### Spiking neuron model

We introduced a stochastic spiking neuron model; each neuron was either excitatory (E) or inhibitory (I). The spikes of each neuron in population P (P = E, I) were generated probabilistically with density *ρ*(*t*), as follows:

$$\begin{eqnarray*}
\rho (t) &= g^\mathrm{P}(u(t)) R(t),
\end{eqnarray*}
$$

where *g*^P^(*u*) denoted a softplus activation intensity function, *u*(*t*) the membrane potential, and *R*(*t*) a refractory factor representing transient suppression of the instantaneous postsynaptic firing rate after a postsynaptic spike. The function $g^\mathrm{P}(u) = r_0^\mathrm{P} \log (1+\exp ((u-u_0 ^\mathrm{P} )/\Delta u^\mathrm{P}))$ with $r_0^\mathrm{E}=1.5$ Hz, $r_0^\mathrm{I}=6.0$ Hz, $u_0^\mathrm{E}=-69.4$ mV, $u_0^\mathrm{I}=-62.5$ mV, and Δ*u*^E^ = Δ*u*^I^ = 0.5 mV. The refractory factor is modeled to be the same for excitatory and inhibitory neurons as $R(t)=(t-\hat{t})^4/(\tau _R^4+(t-\hat{t})^4)$, where $\hat{t}$ denoted the last spike time of the postsynaptic neuron and time constant *τ_R_* = 30 ms. The suppression of this factor after spiking may reflect several mechanisms, including classical refractoriness (64), afterhyperpolarization (AHP) (65), and EPSP suppression by back-propagating action potential (66).

### Single-neuron model

We modeled a postsynaptic neuron that received the feedforward inputs from *N* presynaptic excitatory neurons. Each spike of presynaptic neuron *j* evoked EPSP of amplitude *w_j_* and exponential time course $\epsilon (t)=\exp (-t/\tau _m ^{\mathrm{E}}) H(t)$ with time constant $\tau _m ^{\mathrm{E}} = 25$ ms and Heaviside step function *H*(*t*). The EPSP time course from presynaptic neuron *j* was denoted by $h_j(t) = \sum _f \epsilon (t-t_j^f)$, summing the influence of presynaptic spikes at time $t_j ^{f}$ (*f* = 1, 2, …). Then, the postsynaptic membrane potential *u*(*t*) was denoted by

$$\begin{eqnarray*}
u(t)=u_r + \sum _{j=1} ^{N} w_j h_j (t),
\end{eqnarray*}
$$

with resting membrane potential *u_r_* = −70 mV. Postsynaptic spikes were generated probabilistically with density ρ(*t*) = *g*^E^(*u*(*t*))*R*(*t*), as mentioned in the previous section.

### Information maximizing learning rule

We used the infomax rule for synaptic plasticity (24, 25). The objective function *L* was described as

$$\begin{eqnarray*}
L= I - \lambda \Phi ,
\end{eqnarray*}
$$

with the mutual information term *I*, cost term Φ of the synaptic weights, and coefficient parameter *λ* = 0.32 [1/(mV)^2^]. *I* measures the mutual information between the pre and postsynaptic spike trains. We omitted the homeostatic term included in previous studies because it did not contribute to our results. Each term was denoted by

$$\begin{eqnarray*}
I &= \bigg \langle \log \frac{P(Y \mid X)}{P(Y)} \bigg \rangle _{Y, X}, \\
\Phi &= \frac{1}{2} \sum _j w_j ^2 \langle n_j \rangle _X,
\end{eqnarray*}
$$

with presynaptic and postsynaptic spike trains *X* and *Y*, respectively, and *n_j_* represents the number of presynaptic spikes at synapse *j* during duration *T*. The presynaptic and postsynaptic spike trains up to time *t* were formally denoted by $X(t) = \lbrace x_j (t^{\prime }) = \sum _{f} \delta (t^{\prime }-t_j ^f) \mid j=1, 2, \ldots , N, 0 \le t^{\prime } \lt t \rbrace$ and $Y(t) = \lbrace y (t^{\prime }) = \sum _{f} \delta (t^{\prime }-t_{\mathrm{post}} ^f) \mid 0 \le t^{\prime } \lt t \rbrace$, respectively, where $t_{\mathrm{post}} ^f$ represents the *f*-th (*f* = 1, 2, …) postsynaptic spike timings. We specifically wrote *X* = *X*(*T*) and *Y* = *Y*(*T*) to represent the entire spike train from time *t* = 0 to *t* = *T*. Note that the angular brackets 〈 · 〉_*Y, X*_ and 〈 · 〉_*X*_ represent the averages over all possible *Y, X*, and *X*, respectively.

The optimal synaptic weight change followed the gradient ascent algorithm:

$$\begin{eqnarray*}
\frac{dw_j}{dt} = \alpha \frac{\partial L}{\partial w_j},
\end{eqnarray*}
$$

with a learning rate *α* = 0.01 (mV)^2^. By calculating the gradient (24, 25), the infomax rule was described as

$$\begin{eqnarray*}
\frac{dw_j}{dt}=\alpha \biggl [ C_j(t) B^{\mathrm{post}}(t) - \lambda w_j x_j(t) \biggr ],
\end{eqnarray*}
$$

with

$$\begin{eqnarray*}
C_j(t) &= \lim _{\epsilon \rightarrow +0} \int _0 ^{t+\epsilon } c_j(t^{\prime }) e ^{-\frac{t-t^{\prime }}{\tau _C}} dt^{\prime }, \\
B^{\mathrm{post}} (t) &= y(t) \log \frac{\rho (t)}{\bar{\rho }(t)} -\big ( \rho (t)-\bar{\rho }(t) \big ),
\end{eqnarray*}
$$

described with auxiliary variable $c_j(t)=\left.\frac{\partial \log g^{\mathrm{E}}(u)}{\partial u}\right|_{u=u(t)} \big ( y(t) - \rho (t) \big ) h_j(t)$, expected firing rate $\bar{\rho }(t)=\langle \rho (t) \rangle _{X(t) \mid Y(t)}$, and time constant *τ_C_* = 100 ms. The expected firing rate was further computed as

$$\begin{eqnarray*}
\bar{\rho }(t)=\bar{g}(t) R(t),
\end{eqnarray*}
$$

using the expected intensity $\bar{g}(t)= \langle g^{\mathrm{E}}(u(t)) \rangle _{X(t) \mid Y(t)}$; however, this value can be difficult to calculate if $\bar{g}$ is time dependent. We estimated $\bar{g}$ using slightly different methods for the single-neuron and slow-wave models, as described in the corresponding sections (SI Appendix Methods).

### Theoretical analysis of the STDP effect

For the theoretical analyses in this section, the activation function is not restricted to the softplus function. We evaluated the synaptic changes due to the pre-post stimulation with synchronous presynaptic spikes at *t* = 0 and a postsynaptic spike immediately afterward (at *t* = Δ*t* > 0 with the limit of Δ*t* → 0). To estimate the effect of STDP analytically and qualitatively, we made some simplifications. We approximated that the refractory factor *R* abruptly recovered from zero to one duration *τ_R_* after the postsynaptic spike, and that the spontaneous spiking of the nonstimulated presynaptic neurons yielded a constant baseline membrane potential *u*_0_. We also assumed that, after presynaptic stimulation at *t* = 0, the membrane potential *u*(*t*) = *u*_0_ + ∑_*j* ∈ stim_*w_j_h_j_*(*t*) decayed back to the baseline *u*_0_ by the time the postsynaptic neuron recovered from the refractoriness because $\tau _m^\mathrm{E}$ was smaller than *τ_R_*. Here, stim denotes the set of stimulated neurons. The baseline intensity is denoted by ${\bar{g}}=g^\mathrm{E}(u_0)$, and the peak intensity increase after presynaptic stimulation by $\Delta g = g^\mathrm{E}(u_0+\Delta u)-\bar{g}$, with Δ*u* = ∑_*j* ∈ stim_*w_j_*. In this case, we found $C_j(t) = H(t) \left.\frac{\partial g^{\mathrm{E}}(u)}{\partial u}\right|_{u=u_0+\Delta u}/(\bar{g}+\Delta g)\exp (-t/\tau _C)$ and $B^{\mathrm{post}}(t) = y(t) \log \frac{g^{\mathrm{E}}(u(t))}{\bar{g}}$, using the Heaviside step function *H*(*t*). Note that no extra postsynaptic spike was possible when *h_j_* was significantly positive because the refractory period was longer than that of the stimulus-caused EPSP duration. Hence, the change in the *j*-th synaptic weight due to the STDP stimulus was

$$\begin{eqnarray*}
&&\int C_j(t) B^{\mathrm{post}}(t) dt\\
&&= \left.\frac{\partial g^{\mathrm{E}}(u)}{\partial u}\right|_{u=u_0+\Delta u} \cdot \frac{1}{\bar{g}+\Delta g}\log \left(1+\frac{\Delta g}{\bar{g}}\right).
\end{eqnarray*}
$$

In the case that the activation function is a linear function *g*^E^(*u*) = *g*_0_ · (*u* − *u_r_*), the synaptic change is denoted by $\frac{g_0}{\bar{g}+\Delta g}\log \left(1+\frac{\Delta g}{\bar{g}}\right)$, where Δ*g* does not depend on $\bar{g}$. This result indicated that the increase in synaptic weights due to the information term decreased with the mean firing intensity $\bar{g}$.

### Slow-wave model

We considered *N*^E^ = 800 excitatory neurons, E, and *N*^I^ = 200 inhibitory neurons, I, divided into four local networks. Each local network contained $\frac{N^{\mathrm{E}}}{4}$ excitatory neurons and $\frac{N^{\mathrm{I}}}{4}$ inhibitory neurons. Within each local network, no connections exist between two inhibitory neurons, and all-to-all connections exist between two excitatory neurons as well as between excitatory and inhibitory neurons. Between different local networks, the connection probability from E to E was p_EE_ and the connection probability from E to I was *p*_IE_, whereas inhibitory neurons did not send long-range connections to the other local networks. The connection probabilities *p*_EE_ and *p*_IE_ were set to 0.05 and 0.3, respectively, except for those in Fig. 3C and SI Appendix, Fig. S5. There is no self-coupling in these neurons. The dynamics of the membrane potential $u_i ^\mathrm{P}$ of neuron *i* in population P (P = E, I) were described by

$$\begin{eqnarray*}
\frac{du_i ^{\mathrm{E}}(t)}{dt} &=& -\frac{u_i ^{\mathrm{E}}(t)-u_r}{\tau _m ^\mathrm{E}} + \sum \limits _{j=1} ^{N^\mathrm{E} } w_{ij} ^{\mathrm{EE}} S_j ^\mathrm{E}(t)+ \sum\limits _{j=1} ^{N^\mathrm{I} } w_{ij} ^{\mathrm{EI}} S_j ^\mathrm{I}(t)\\
&& \quad + I _i ^{\mathrm{ext}} (t) -I _i ^{a} (t),\\
\frac{du_i ^{\mathrm{I}}(t)}{dt} &=& -\frac{u_i ^{\mathrm{I}}(t)-u_r}{\tau _m ^\mathrm{I}} + \sum\limits _{j=1} ^{N^\mathrm{E} } w_{ij} ^{\mathrm{IE}} S_j ^\mathrm{E}(t),
\end{eqnarray*}
$$

with the recurrent synaptic weight $w_{ij} ^{\mathrm{EE}}$, $w_{ij} ^{\mathrm{EI}}$, and $w_{ij} ^{\mathrm{IE}}$ from excitatory neuron *j* to excitatory neuron *i*, from inhibitory neuron *j* to excitatory neuron *i*, and from excitatory neuron *j* to inhibitory neuron *i*, respectively, membrane time constant $\tau _m ^{\mathrm{E}} = 25$ ms for excitatory neurons and $\tau _m ^{\mathrm{I}} = 5$ ms for inhibitory neurons, the resting membrane potential *u_r_* = −70 mV, spike train $S_j ^\mathrm{E} (t) = \sum _f \delta (t-t_j ^{f})$ or $S_j ^\mathrm{I} (t) = \sum _f \delta (t-t_j ^{f})$ of excitatory or inhibitory neuron *j* described with its *f*-th spike time $t_j ^{f}$, external current $I _i ^{\mathrm{ext}} (t)$, and adaptation current $I _i ^{a} (t)$. The spikes of neuron *i* in population P (P = E, I) were generated probabilistically with an instantaneous firing rate $g^{\mathrm{P}}(u_i ^\mathrm{P}(t)) R_i(t)$, as mentioned in the previous section, with its refractory factor *R_i_*. The recurrent synaptic weight was fixed at $w_{ij} ^{\mathrm{EE}} = 0.16$ mV, $w_{ij} ^{\mathrm{EI}} = -0.14$ mV, and $w_{ij} ^{\mathrm{IE}} = 0.66$ mV if synaptic connections existed from neuron *j* to neuron *i*, whereas it was set to 0 mV if there were no synaptic connections. The external current was a feedforward input only to the excitatory neuron *1*, described as $I _i ^{\mathrm{ext}} (t) = \delta _{i1} \sum _{j=1} ^{N^{\mathrm{ext}}} w_j ^{\mathrm{ext}} S_j^{\mathrm{ext}}(t)$, with the Kronecker delta δ_*i*1_, synaptic weights $w_j ^{\mathrm{ext}}$ from *N*^ext^ presynaptic neurons, and presynaptic spike train $S_j^{\mathrm{ext}}(t) = \sum _{t_j ^f} \delta (t-t_j ^f)$, where $t_j ^f$ represented the spike timing. Presynaptic neurons emitted the synchronous spikes upon STDP stimulation (Fig. 4) or task stimulation (Fig. 5 and SI Appendix, Fig. S7), although they did not emit spontaneous spikes. The STDP and task simulations (Figs. 4 and 5 and SI Appendix, Fig. S7) changed the feedforward synaptic weights *w_j_* according to the infomax rule. The dynamics of the adaptation current $I _i ^{a} (t)$ of neuron *i* were described as follows

$$\begin{eqnarray*}
\frac{d I _i ^{a} (t)}{dt} = -\frac{I _i ^{a} (t)}{\tau _{a}} + \beta S_i ^\mathrm{E}(t),
\end{eqnarray*}
$$

with a time constant of *τ_a_* = 1,500 ms and a constant value of *β* = 0.0077 mV/ms.

### Stage classification

First, we classified the up and down states of each population using two transition thresholds. When the mean membrane potential of excitatory neurons in each local network exceeded the up-transition threshold *θ*_up_ = −68.25 mV, this moment was judged as a state transition to the up state. When the mean membrane potential fell below the down-transition threshold *θ*_down_ = −69.75 mV, this moment was judged as a state transition to the down state. We then classified each state into the global or local states. When a local network was in the down state, it was classified into the global down state if the number of other local networks in the down states was two or three, while it was classified as the local down state otherwise. Likewise, when a focused population was in the up state, it was classified into the global up state if the number of other local networks in the up states was two or three, while it was classified as the local up state otherwise.

### Simulation environment

All numerical calculations were performed using the custom-written Python codes. The model was simulated in discrete time with time steps of 1 ms. Synaptic weights did not change during the first 10,000 ms (200,000 ms in Fig. 5 and SI Appendix, Fig. S7) in all simulations to avoid the effects of initial values. The initial value of the last spike time was set to −10,000 ms for all neurons.

## Supplementary Material

pgac286_Supplemental_File

## Data Availability

The codes used for simulations are available at https://github.com/kkyoshida/SleepInfomax.

## Preprint

A preprint version of the manuscript has been posted on https://www.biorxiv.org/content/10.1101/2022.03.22.485283v1.

## References

[1] Diekelmann S , Born J. 2010. The memory function of sleep. Nat Rev Neurosci. 11:114–126. 20046194 10.1038/nrn2762

[2] Klinzing JG , Niethard N, Born J. 2019. Mechanisms of systems memory consolidation during sleep. Nat Neurosci. 22:1598–1610. 31451802 10.1038/s41593-019-0467-3

[3] Tononi G , Cirelli C. 2014. Sleep and the price of plasticity: from synaptic and cellular homeostasis to memory consolidation and integration. Neuron. 81:12–34. 24411729 10.1016/j.neuron.2013.12.025 PMC3921176

[4] Steriade M , Timofeev I, Grenier F. 2001. Natural waking and sleep states: a view from inside neocortical neurons. J Neurophysiol. 85:1969–1985. 11353014 10.1152/jn.2001.85.5.1969

[5] Huber R , Ghilardi MF, Massimini M, Tononi G. 2004. Local sleep and learning. Nature. 430:78–81. 15184907 10.1038/nature02663

[6] Marshall L , Helgadóttir H, Mölle M, Born J. 2006. Boosting slow oscillations during sleep potentiates memory. Nature. 444:610–613. 17086200 10.1038/nature05278

[7] Miyamoto D , et al. 2016. Top-down cortical input during NREM sleep consolidates perceptual memory. Science. 352:1315–1318. 27229145 10.1126/science.aaf0902

[8] Miyamoto D , Hirai D, Murayama M. 2017. The roles of cortical slow waves in synaptic plasticity and memory consolidation. Front Neural Circ. 11:1–8. 10.3389/fncir.2017.00092 PMC5703076 29213231

[9] Ngo HVV , Martinetz T, Born J, Mölle M. 2013. Auditory closed-loop stimulation of the sleep slow oscillation enhances memory. Neuron. 78:545–553. 23583623 10.1016/j.neuron.2013.03.006

[10] Bernardi G , Siclari F, Handjaras G, Riedner BA, Tononi G. 2018. Local and widespread slow waves in stable NREM sleep: evidence for distinct regulation mechanisms. Front Hum Neurosci. 12:1–13. 29970995 10.3389/fnhum.2018.00248 PMC6018150

[11] Dang-Vu TT , et al., 2008. Spontaneous neural activity during human slow wave sleep. Proc Natl Acad Sci USA. 105:15160–15165. 18815373 10.1073/pnas.0801819105 PMC2567508

[12] Genzel L , Kroes MCW, Dresler M, Battaglia FP. 2014. Light sleep versus slow wave sleep in memory consolidation: a question of global versus local processes?. Trends Neurosci. 37:10–19. 24210928 10.1016/j.tins.2013.10.002

[13] Kim J , Gulati T, Ganguly K. 2019. Competing roles of slow oscillations and delta waves in memory consolidation versus forgetting. Cell. 179:514–526. 31585085 10.1016/j.cell.2019.08.040 PMC6779327

[14] Siclari F et al. 2014. Two distinct synchronization processes in the transition to sleep: a high-density electroencephalographic study. Sleep. 37:1621–1637F. 25197810 10.5665/sleep.4070 PMC4173919

[15] Steriade M , McCormick DA, Sejnowski TJ. 1993. Thalamocortical oscillations in the sleeping and aroused brain. Science. 262:679–685. 8235588 10.1126/science.8235588

[16] Steriade M , Nunez A, Amzica F. 1993. Intracellular analysis of relations between the slow (< 1 Hz) neocortical oscillation and other sleep rhythms of the electroencephalogram. J Neurosci. 13:3266–3283. 8340807 10.1523/JNEUROSCI.13-08-03266.1993 PMC6576520

[17] Gulati T , Ramanathan DS, Wong CC, Ganguly K. 2014. Reactivation of emergent task-related ensembles during slow-wave sleep after neuroprosthetic learning. Nat Neurosci. 17: 1107–1113. 24997761 10.1038/nn.3759 PMC5568667

[18] Peyrache A , Khamassi M, Benchenane K, Wiener SI, Battaglia FP. 2009. Replay of rule-learning related neural patterns in the prefrontal cortex during sleep. Nat Neurosci. 12:919–926. 19483687 10.1038/nn.2337

[19] Ji D , Wilson MA. 2007. Coordinated memory replay in the visual cortex and hippocampus during sleep. Nat Neurosci. 10:100–107. 17173043 10.1038/nn1825

[20] González-Rueda A , Pedrosa V, Feord RC, Clopath C, Paulsen O. 2018. Activity-dependent downscaling of subthreshold synaptic inputs during slow-wave-sleep-like activity in vivo. Neuron. 97:1244–1252. 29503184 10.1016/j.neuron.2018.01.047 PMC5873548

[21] Bartram J , et al. 2017. Cortical up states induce the selective weakening of subthreshold synaptic inputs. Nat Commun. 8:665. 28939859 10.1038/s41467-017-00748-5 PMC5610171

[22] Barlow HB . 1961. Possible principles underlying the transformations of sensory messages. In Rosenblith W, editor. Sensory communication, Cambridge (MA): MIT Press. p. 217–234.

[23] Linsker R . 1988. Self-organization in a perceptual network. Computer. 21:105–117.

[24] Toyoizumi T , Pfister JP, Aihara K, Gerstner W. 2005. Generalized Bienenstock–Cooper–Munro rule for spiking neurons that maximizes information transmission. Proc Natl Acad Sci USA. 102:5239–5244. 15795376 10.1073/pnas.0500495102 PMC555686

[25] Toyoizumi T , Pfister JP, Aihara K, Gerstner W. 2007. Optimality model of unsupervised spike-timing-dependent plasticity: synaptic memory and weight distribution. Neural Comput. 19:639–671. 17298228 10.1162/neco.2007.19.3.639

[26] Jercog D , et al. 2017. UP-DOWN cortical dynamics reflect state transitions in a bistable network. eLife. 6:1–33. 10.7554/eLife.22425 PMC5582872 28826485

[27] Bazhenov M , Timofeev I, Steriade M, Sejnowski TJ. 2002. Model of thalamocortical slow-wave sleep oscillations and transitions to activated states. J Neurosci. 22:8691–8704. 12351744 10.1523/JNEUROSCI.22-19-08691.2002 PMC6757797

[28] Compte A , Sanchez-Vives MV, McCormick DA, Wang XJ. 2003. Cellular and network mechanisms of slow oscillatory activity (<1 Hz) and wave propagations in a cortical network model. J Neurophysiol. 89:2707–2725. 12612051 10.1152/jn.00845.2002

[29] Tatsuki F , Sunagawa GA, Shi S, Susaki EA, Yukinaga H, Perrin D, et al. 2016. Involvement of Ca^2+^-dependent hyperpolarization in sleep duration in mammals. Neuron. 90:70–85. 26996081 10.1016/j.neuron.2016.02.032

[30] Yoshida K , et al. 2018. Leak potassium channels regulate sleep duration. Proc Natl Acad Sci USA. 115:E9459–E9468. 30224462 10.1073/pnas.1806486115 PMC6176580

[31] Rubin DB , VanHooser SD, Miller KD. 2015. The stabilized supralinear network: a unifying circuit motif underlying multi-input integration in sensory cortex. Neuron. 85:402–417. 25611511 10.1016/j.neuron.2014.12.026 PMC4344127

[32] Tsodyks MV , Skaggs WE, Sejnowski TJ, McNaughton BL. 1997. Paradoxical effects of external modulation of inhibitory interneurons. J Neurosci. 17:4382–4388. 9151754 10.1523/JNEUROSCI.17-11-04382.1997 PMC6573545

[33] Ozeki H , Finn IM, Schaffer ES, Miller KD, Ferster D. 2009. Inhibitory stabilization of the cortical network underlies visual surround suppression. Neuron. 62:578–592. 19477158 10.1016/j.neuron.2009.03.028 PMC2691725

[34] Latchoumane CFV , Ngo HVV, Born J, Shin HS. 2017. Thalamic spindles promote memory formation during sleep through triple phase-locking of cortical, thalamic, and hippocampal rhythms. Neuron. 95:424–435. 28689981 10.1016/j.neuron.2017.06.025

[35] Peyrache A , Seibt J. 2020. A mechanism for learning with sleep spindles. Philos Trans R Soc Lond B Biol Sci. 375:20190230. 32248788 10.1098/rstb.2019.0230 PMC7209910

[36] Niethard N , Ngo HVV, Ehrlich I, Born J. 2018. Cortical circuit activity underlying sleep slow oscillations and spindles. Proc Natl Acad Sci USA. 115:E9220–E9229. 30209214 10.1073/pnas.1805517115 PMC6166829

[37] Seibt J , et al. 2017. Cortical dendritic activity correlates with spindle-rich oscillations during sleep in rodents. Nat Commun. 8:684. 28947770 10.1038/s41467-017-00735-w PMC5612962

[38] Rosanova M , Ulrich D. 2005. Pattern-specific associative long-term potentiation induced by a sleep spindle-related spike train. J Neurosci. 25:9398–405. 16221848 10.1523/JNEUROSCI.2149-05.2005 PMC6725710

[39] Gulati T , Guo L, Ramanathan DS, Bodepudi A, Ganguly K. 2017. Neural reactivations during sleep determine network credit assignment. Nat Neurosci. 20:1277–1284. 28692062 10.1038/nn.4601 PMC5808917

[40] Todorova R , Zugaro M. 2019. Isolated cortical computations during delta waves support memory consolidation. Science. 366:377–381. 31624215 10.1126/science.aay0616

[41] Yang G , et al. 2014. Sleep promotes branch-specific formation of dendritic spines after learning. Science. 344:1173–1178. 24904169 10.1126/science.1249098 PMC4447313

[42] Durkin J , Aton SJ. 2016. Sleep-dependent potentiation in the visual system is at odds with the synaptic homeostasis hypothesis. Sleep. 39:155–159. 26285006 10.5665/sleep.5338 PMC4678346

[43] Goto A , et al. 2021. Stepwise synaptic plasticity events drive the early phase of memory consolidation. Science. 374:857–863. 34762472 10.1126/science.abj9195

[44] Chauvette S , Seigneur J, Timofeev I. 2012. Sleep oscillations in the thalamocortical system induce long-term neuronal plasticity. Neuron. 75:1105–1113. 22998877 10.1016/j.neuron.2012.08.034 PMC3458311

[45] Timofeev I , Chauvette S. 2017. Sleep slow oscillation and plasticity. Curr Opin Neurobiol. 44:116–126. 28453998 10.1016/j.conb.2017.03.019

[46] Timofeev I , Chauvette S. 2018. Sleep, anesthesia, and plasticity. Neuron. 97:1200–1202. 29566787 10.1016/j.neuron.2018.03.013

[47] Puentes-Mestril C , Aton SJ. 2017. Linking network activity to synaptic plasticity during sleep: hypotheses and recent data. Front Neural Circ. 11:1–18. 10.3389/fncir.2017.00061 PMC5592216 28932187

[48] Hayama T , et al. 2013. GABA promotes the competitive selection of dendritic spines by controlling local Ca^2+^ signaling. Nat Neurosci. 16:1409–1416. 23974706 10.1038/nn.3496 PMC4135703

[49] De Pittà M , Brunel N, Volterra A. 2016. Astrocytes: orchestrating synaptic plasticity?. Neuroscience. 323:43–61. 25862587 10.1016/j.neuroscience.2015.04.001

[50] Vyazovskiy VV , Harris KD. 2013. Sleep and the single neuron: the role of global slow oscillations in individual cell rest. Nat Rev Neurosci. 14:443–451. 23635871 10.1038/nrn3494 PMC3972489

[51] Valero M , et al. 2021. Sleep down state-active ID2/Nkx2.1 interneurons in the neocortex. Nat Neurosci. 24:401–411. 33619404 10.1038/s41593-021-00797-6 PMC9662703

[52] Wei Y , Krishnan GP, Bazhenov M. 2016. Synaptic mechanisms of memory consolidation during sleep slow oscillations. J Neurosci. 36:4231–4247. 27076422 10.1523/JNEUROSCI.3648-15.2016 PMC4829648

[53] Wei Y , Krishnan GP, Komarov M, Bazhenov M. 2018. Differential roles of sleep spindles and sleep slow oscillations in memory consolidation. PLOS Comput Biol. 14:e1006322. 29985966 10.1371/journal.pcbi.1006322 PMC6053241

[54] Timofeev I , Schoch SF, LeBourgeois MK, Huber R, Riedner BA, Kurth S. 2020. Spatio-temporal properties of sleep slow waves and implications for development. Curr Opin Physiol. 15:172–182. 32455180 10.1016/j.cophys.2020.01.007 PMC7243595

[55] Steriade M , Timofeev I. 2003. Neuronal plasticity in thalamocortical networks during sleep and waking oscillations. Neuron. 37:563–76. 12597855 10.1016/s0896-6273(03)00065-5

[56] Hay YA , et al. 2021. Thalamus mediates neocortical down state transition via GABAB-receptor-targeting interneurons. Neuron. 109:2682–2690. 34314698 10.1016/j.neuron.2021.06.030

[57] Narikiyo K , et al. 2020. The claustrum coordinates cortical slow-wave activity. Nat Neurosci. 23:741–753. 32393895 10.1038/s41593-020-0625-7

[58] Joo HR , Frank LM. 2018. The hippocampal sharp wave–ripple in memory retrieval for immediate use and consolidation. Nat Rev Neurosci. 19:744–757. 30356103 10.1038/s41583-018-0077-1 PMC6794196

[59] Sadeh S , Clopath C. 2021. Inhibitory stabilization and cortical computation. Nat Rev Neurosci. 22:21–37. 33177630 10.1038/s41583-020-00390-z

[60] Sanzeni A , et al. 2020. Inhibition stabilization is a widespread property of cortical networks. eLife. 9:1–39. 10.7554/eLife.54875 PMC7324160 32598278

[61] Steinmetz NA , et al. 2021. Neuropixels 2.0: a miniaturized high-density probe for stable, long-term brain recordings. Science. 372(6539):eabf4588. 33859006 10.1126/science.abf4588 PMC8244810

[62] Landmann N , et al. 2014. The reorganisation of memory during sleep. Sleep Med Rev. 18:531–541. 24813468 10.1016/j.smrv.2014.03.005

[63] Deperrois N , Petrovici MA, Senn W, Jordan J. 2022. Learning cortical representations through perturbed and adversarial dreaming. eLife. 11:1–34. 10.7554/eLife.76384 PMC9071267 35384841

[64] Fuortes MGF , Mantegazzini F. 1962. Interpretation of the repetitive firing of nerve cells. J Gen Physiol. 45:1163–1179. 13895926 10.1085/jgp.45.6.1163 PMC2195242

[65] Sah P , Faber ESL. 2002. Channels underlying neuronal calcium-activated potassium currents. Prog Neurobiol. 66:345–353. 12015199 10.1016/s0301-0082(02)00004-7

[66] Froemke RC , Poo MM, Dan Y. 2005. Spike-timing-dependent synaptic plasticity depends on dendritic location. Nature. 434:221–225. 15759002 10.1038/nature03366

