# Supplementary material for: Information maximization explains state-dependent synaptic plasticity and memory reorganization during non-rapid eye movement sleep
Source: PNAS Nexus. 2022 Dec 10;2(1):pgac286. doi: 10.1093/pnasnexus/pgac286 (PMC9833047; doi:10.1093/pnasnexus/pgac286)
Supplement: pgac286_Supplemental_File [file pgac286_supplemental_file.pdf]

Supporting Information for

Information maximization explains state-dependent synaptic plasticity and memory reorganization during non-rapid eye movement sleep

Kensuke Yoshida and Taro Toyoizumi

Kensuke Yoshida and Taro Toyoizumi.
E-mail: kensuke_yoshida@mist.i.u-tokyo.ac.jp, taro.toyoizumi@riken.jp.

This PDF file includes:

Supporting text
Figs. S1 to S7
SI References

Supporting Information Text

Methods

Simulation of STDP in the single-neuron model. We considered two populations of presynaptic neurons projecting onto a postsynaptic neuron. One population represented the stimulated neurons, while the other represented non-stimulated neurons. $N_1 = 100$ non-stimulated neurons had fixed synaptic weight $w_j = 0.5$ mV and emitted spikes at $\rho_{\mathrm{pre}}^{\mathrm{down}} = 0.1$ Hz in down state and $\rho_{\mathrm{pre}}^{\mathrm{up}} = 2.0$ Hz in up states. $N_2 = 20$ stimulated neurons had a plastic synaptic weight, which was initialized at $w_j = 0.5$ mV and updated by the infomax rule, and spiked only when they were stimulated. In Figs. S3 and S4, we varied the N_2 value and the initial value of w_j in the stimulated neurons. STDP stimulation with time interval Δt evoked spikes in all the stimulated neurons at time 0 and evoked a postsynaptic spike at time Δt. The STDP stimulations were given twice 5000 ms apart. The amplitudes of artificial postsynaptic depolarization in down states and hyperpolarization in up states in Fig. S2 were set to $(\rho_{\mathrm{pre}}^{\mathrm{up}} - \rho_{\mathrm{pre}}^{\mathrm{down}}) \cdot N_1 w_j \tau_m^{\mathrm{E}}$, the expected difference between down and up states.

In this model, $\bar{g}$ was estimated by averaging $g^{\mathrm{E}}(u(t))$ over fluctuating $u(t)$, while the stimulated presynaptic neurons remained silent and the non-stimulated presynaptic neurons spontaneously generated Poisson spikes at different firing rates for the up or down states.

Theoretical analysis in various activation functions. We considered the case in which the activation function is not linear, using the theoretical result in the main text. With the limit of $\Delta u \to +0$, the change in the j-th synaptic weight was approximated by

$$\int C_j(t) B^{\mathrm{post}}(t) dt \simeq \left(\frac{\left.\frac{\partial g^{\mathrm{E}}(u)}{\partial u}\right|_{u=u_0}}{g^{\mathrm{E}}(u_0)} \right)^2 \Delta u.$$

Hence, to have decreasing synaptic changes due to the information term as the mean firing intensity increases is that the function $f(u_0) = (\left.\frac{\partial g^{\mathrm{E}}(u)}{\partial u}\right|_{u=u_0} / g^{\mathrm{E}}(u_0))^2$ is monotonically decreasing with u_0. This condition holds true for typical activation functions such as the softplus function and the power function $g^E(u) = k_p [u - u_{\theta_p}]_+^a$ with $k_p > 0$, $a > 0$, $u > u_{\theta_p}$, and the rectified linear function $[\cdot]_+$, but not for the exponential activation function $g^E(u) = k_e \cdot \exp(u - u_{\theta_e})$, as shown in Fig. S1 (see the next section for the analytical proof). We also showed that the condition holds true for the response function to noisy input currents in a leaky integrate-and-fire neuron $g^E(u) = \left(\tau_m^E \sqrt{\pi} \int_{\frac{u_{\theta_0} - u}{\sigma}}^{\frac{u_{\theta_1} - u}{\sigma}} \exp(x^2)[1 + \mathrm{erf}(x)] dx \right)^{-1}$ (1) with representative parameters $u_{\theta_0} = -70.0$ mV, $u_{\theta_1} = -64.0$ mV, and $\sigma = 2.0$ mV in Fig. S1. Although some literature used the exponential activation function (2), other experimental studies used the power (3) or softplus-like (4, 5) activation function. We adopted the softplus activation function in this study as a representative.

Analytical proof of the previous section. In this section, we prove that the function $f(u) = (\frac{\partial g^{\mathrm{E}}(u)}{\partial u} / g^{\mathrm{E}}(u))^2$ is monotonically decreasing for the softplus function and power function, but not for the exponential function.

First, we consider the softplus function $g^{\mathrm{E}}(u) = r_0^{\mathrm{E}} \log(1 + \exp((u - u_0^{\mathrm{E}})/\Delta u^{\mathrm{E}}))$ with $r_0^{\mathrm{E}} > 0$ and $\Delta u^{\mathrm{E}} > 0$. The function $f(u)$ and $\frac{\partial f(u)}{\partial u}$ is described as follows:

$$f(u) = \left(\frac{1}{\Delta u^{\mathrm{E}}} \cdot \frac{\exp((u - u_0^{\mathrm{E}})/\Delta u^{\mathrm{E}})}{1 + \exp((u - u_0^{\mathrm{E}})/\Delta u^{\mathrm{E}})} \cdot \frac{1}{\log(1 + \exp((u - u_0^{\mathrm{E}})/\Delta u^{\mathrm{E}}))} \right)^2,$$

$$\frac{\partial f(u)}{\partial u} = \frac{2 f(u)}{\Delta u^{\mathrm{E}}(1 + \exp((u - u_0^{\mathrm{E}})/\Delta u^{\mathrm{E}})) \cdot \log(1 + \exp((u - u_0^{\mathrm{E}})/\Delta u^{\mathrm{E}}))} \cdot \left(\log(1 + \exp((u - u_0^{\mathrm{E}})/\Delta u^{\mathrm{E}})) - \exp((u - u_0^{\mathrm{E}})/\Delta u^{\mathrm{E}}) \right).$$

Since $\frac{\partial f(u)}{\partial u} < 0$ for all u, $f(u)$ is monotonically decreasing.

Second, we consider the power function $g^E(u) = k_p [u - u_{\theta_p}]_+^a$ with $a > 0$. The function $f(u)$ in $u > u_{\theta_p}$ is described as

$$f(u) = \frac{a^2}{(u - u_{\theta_p})^2},$$

which indicates that $f(u)$ is monotonically decreasing.

Finally, we consider the exponential activation function $g^E(u) = k_e \cdot \exp(u - u_{\theta_e})$. In this case, $f(u) = 1$ for all u, not monotonically decreasing.

Phase plane analysis. Assuming a large population of neurons in each local network, the average dynamics of the membrane potential u^{P} ($\mathrm{P}=\mathrm{E},\mathrm{I}$) in the population P (containing $\frac{N^{\mathrm{P}}}{4}$ neurons) was described by

$$\frac{du^{\mathrm{E}}}{dt} = -\frac{u^{\mathrm{E}}-u_r}{\tau_m^{\mathrm{E}}} + (\frac{N^{\mathrm{E}}}{4}-1)w^{\mathrm{EE}}g^{\mathrm{E}}(u^{\mathrm{E}}) + \frac{N^{\mathrm{I}}}{4}w^{\mathrm{EI}}g^{\mathrm{I}}(u^{\mathrm{I}}) + I^{\mathrm{EE}} - I^a,$$
$$\frac{du^{\mathrm{I}}}{dt} = -\frac{u^{\mathrm{I}}-u_r}{\tau_m^{\mathrm{I}}} + \frac{N^{\mathrm{E}}}{4}w^{\mathrm{IE}}g^{\mathrm{E}}(u^{\mathrm{E}}) + I^{\mathrm{IE}},$$

with the synaptic weight $w^{\mathrm{EE}}=0.16$ mV, $w^{\mathrm{EI}}=-0.14$ mV, $w^{\mathrm{IE}}=0.66$ mV, and I^{PE} denoting the long-range excitatory currents to population P from the other local networks. External inputs were not considered here. We denoted by $I^{\mathrm{PE}} = \frac{3}{4}N^{\mathrm{E}}p_{\mathrm{PE}}w^{\mathrm{PE}}r_{\mathrm{other}}$ the excitatory input from the other local networks to population P, where r_{other} represented the firing rate of the excitatory populations in the other local networks. r_{other} was set to 0 Hz and 6 Hz when the other local networks were in the down and up states, respectively. The adaptation current I^a was set to 0, 0.01, 0.05, or 0.18 mV/ms.

Furthermore, we conducted a stability analysis in the slow-wave model. We approximated the activation function $g^{\mathrm{P}}(u)$ ($\mathrm{P}=\mathrm{E},\mathrm{I}$) using the threshold-linear function $g^{\mathrm{P}}(u)=g_0^{\mathrm{P}}[u-u_0^{\mathrm{P}}]_+$, where $g_0^{\mathrm{P}}=r_0^{\mathrm{P}}/\Delta u^{\mathrm{P}}$ and $[x]_+$ took the value x if $x>0$ and zero otherwise. (Note that the phase plane plots in Figs. 3*G* and S5*B* are depicted using the original softplus activation function.) By using this approximation, the differential equations above were described as

$$\frac{du^{\mathrm{E}}}{dt} = -\frac{u^{\mathrm{E}}-u_r}{\tau_m^{\mathrm{E}}} + W^{\mathrm{EE}}g_0^{\mathrm{E}}[u^{\mathrm{E}}-u_0^{\mathrm{E}}]_+ + W^{\mathrm{EI}}g_0^{\mathrm{I}}[u^{\mathrm{I}}-u_0^{\mathrm{I}}]_+ + I^{\mathrm{EE}} - I^a, \quad [1]$$
$$\frac{du^{\mathrm{I}}}{dt} = -\frac{u^{\mathrm{I}}-u_r}{\tau_m^{\mathrm{I}}} + W^{\mathrm{IE}}g_0^{\mathrm{E}}[u^{\mathrm{E}}-u_0^{\mathrm{E}}]_+ + I^{\mathrm{IE}}, \quad [2]$$

where $W^{\mathrm{EE}}=(\frac{N^{\mathrm{E}}}{4}-1)w^{\mathrm{EE}}$, $W^{\mathrm{EI}}=\frac{N^{\mathrm{I}}}{4}w^{\mathrm{EI}}$, and $W^{\mathrm{IE}}=\frac{N^{\mathrm{E}}}{4}w^{\mathrm{IE}}$.

We first investigated the case in which the external currents I^{EE} and I^{IE} were zero. We searched the condition that both the down state satisfying $u^{\mathrm{E}}<u_0^{\mathrm{E}}$ and $u^{\mathrm{I}}<u_0^{\mathrm{I}}$ and the up state satisfying $u^{\mathrm{E}}>u_0^{\mathrm{E}}$ and $u^{\mathrm{I}}>u_0^{\mathrm{I}}$ existed stably.

If a solution satisfied $u^{\mathrm{E}}<u_0^{\mathrm{E}}$ and $u^{\mathrm{I}}<u_0^{\mathrm{I}}$, then the fixed point was $(u^{\mathrm{E}},u^{\mathrm{I}})=(u_r-\tau_m^{\mathrm{E}}I^a, u_r)$. Hence, this solution existed when it satisfied

$$u_r-\tau_m^{\mathrm{E}}I^a < u_0^{\mathrm{E}}, \quad [3]$$
$$u_r < u_0^{\mathrm{I}}. \quad [4]$$

The Jacobian matrix J of differential equations [1] and [2] at the fixed point satisfying $u^{\mathrm{E}}<u_0^{\mathrm{E}}$ and $u^{\mathrm{I}}<u_0^{\mathrm{I}}$ was described as

$$J = \begin{pmatrix} -\frac{1}{\tau_m^{\mathrm{E}}} & 0 \\ 0 & -\frac{1}{\tau_m^{\mathrm{I}}} \end{pmatrix}.$$

Because all eigenvalues of the Jacobian matrix J had a negative real part, the fixed point was stable.

If a solution satisfied $u^{\mathrm{E}}>u_0^{\mathrm{E}}$ and $u^{\mathrm{I}}>u_0^{\mathrm{I}}$, then the fixed point of the equations was

$$u^{\mathrm{E}} = u_0^{\mathrm{E}} + \frac{1}{M}\left(-W^{\mathrm{EI}}g_0^{\mathrm{I}}(u_0^{\mathrm{I}}-u_r) - \frac{u_0^{\mathrm{E}}-u_r}{\tau_m^{\mathrm{E}}} - I^a\right),$$
$$u^{\mathrm{I}} = u_r + \frac{\tau_m^{\mathrm{I}}W^{\mathrm{IE}}g_0^{\mathrm{E}}}{M}\left(-W^{\mathrm{EI}}g_0^{\mathrm{I}}(u_0^{\mathrm{I}}-u_r) - \frac{u_0^{\mathrm{E}}-u_r}{\tau_m^{\mathrm{E}}} - I^a\right),$$

where $M=\frac{1}{\tau_m^{\mathrm{E}}}-(W^{\mathrm{EE}}+W^{\mathrm{EI}}W^{\mathrm{IE}}g_0^{\mathrm{I}}\tau_m^{\mathrm{I}})g_0^{\mathrm{E}}$. This solution existed when $u^{\mathrm{E}}>u_0^{\mathrm{E}}$ and $u^{\mathrm{I}}>u_0^{\mathrm{I}}$ were satisfied. Under the condition [4], it was equivalent to

$$\frac{\tau_m^{\mathrm{I}}W^{\mathrm{IE}}g_0^{\mathrm{E}}}{M}\left(I^a+\frac{u_0^{\mathrm{E}}-u_r}{\tau_m^{\mathrm{E}}}\right) < \frac{1}{M}\left(W^{\mathrm{EE}}g_0^{\mathrm{E}}-\frac{1}{\tau_m^{\mathrm{E}}}\right)(u_0^{\mathrm{I}}-u_r). \quad [5]$$

The Jacobian matrix J of differential equations [1] and [2] at the fixed point satisfying $u^{\mathrm{E}}>u_0^{\mathrm{E}}$ and $u^{\mathrm{I}}>u_0^{\mathrm{I}}$ was described as

$$J = \begin{pmatrix} -\frac{1}{\tau_m^{\mathrm{E}}}+W^{\mathrm{EE}}g_0^{\mathrm{E}} & W^{\mathrm{EI}}g_0^{\mathrm{I}} \\ W^{\mathrm{IE}}g_0^{\mathrm{E}} & -\frac{1}{\tau_m^{\mathrm{I}}} \end{pmatrix}.$$

If all eigenvalues of the Jacobian matrix J had a negative real part, then the fixed point was stable. This condition was equivalent to having a negative trace and a positive determinant of matrix J; namely,

$$-\frac{1}{\tau_m^{\mathrm{E}}}-\frac{1}{\tau_m^{\mathrm{I}}}+W^{\mathrm{EE}}g_0^{\mathrm{E}} < 0, \quad [6]$$
$$M > 0. \quad [7]$$

In summary, two stable states existed when the conditions [3]–[7] were satisfied.

Next, we considered the case where the external currents I^{EE} and I^{IE} were not zero. We investigated the condition in which the long-range excitatory input from the other local networks suppressed the firing rates of the local excitatory population in the up state. The fixed point corresponding to the up state was

$$u^{\mathrm{E}} = u^{\mathrm{E}}_{\mathrm{local}} + \frac{1}{M}(I^{\mathrm{EE}} + W^{\mathrm{EI}} g_0^{\mathrm{I}} \tau_m^{\mathrm{I}} I^{\mathrm{IE}}),$$

$$u^{\mathrm{I}} = u^{\mathrm{I}}_{\mathrm{local}} + \frac{W^{\mathrm{IE}} g_0^{\mathrm{E}} \tau_m^{\mathrm{I}}}{M}(I^{\mathrm{EE}} + W^{\mathrm{EI}} g_0^{\mathrm{I}} \tau_m^{\mathrm{I}} I^{\mathrm{IE}}) + \tau_m^{\mathrm{I}} I^{\mathrm{IE}},$$

where $u^{\mathrm{P}}_{\mathrm{local}}$ $(\mathrm{P} = \mathrm{E}, \mathrm{I})$ was a fixed point when external currents I^{EE} and I^{IE} were zero. Hence, the condition in which the external currents suppressed the excitatory firing rates was described as:

$$I^{\mathrm{EE}} + W^{\mathrm{EI}} g_0^{\mathrm{I}} \tau_m^{\mathrm{I}} I^{\mathrm{IE}} < 0.$$

Using $I^{\mathrm{PE}} = \frac{3}{4} N^{\mathrm{E}} p_{\mathrm{PE}} w^{\mathrm{PE}} r_{\mathrm{other}}$, the condition was further transformed into:

$$p_{\mathrm{EE}} w^{\mathrm{EE}} + W^{\mathrm{EI}} g_0^{\mathrm{I}} \tau_m^{\mathrm{I}} p_{\mathrm{IE}} w^{\mathrm{IE}} < 0.$$

Simulation of STDP in the slow-wave model. In the slow-wave model, the expected firing intensity $\bar{g}(t)$ was estimated by the population-mean firing intensity of the excitatory neurons in the same population, described as

$$\bar{g}(t) = \frac{1}{\frac{N^{\mathrm{E}}}{4}} \sum_{i=1}^{\frac{N^{\mathrm{E}}}{4}} g^{\mathrm{E}}(u_i^{\mathrm{E}}(t)),$$

where the excitatory neurons i $(i = 1, 2, \ldots, \frac{N^{\mathrm{E}}}{4})$ were in the same excitatory population (E1) as the postsynaptic neuron in consideration. In Fig. 4, we considered $N^{\mathrm{ext}} = 20$ or 40 presynaptic neurons that had the plastic synaptic weights with an initial value of $w_j^{\mathrm{ext}} = 0.5$ or 0.09 mV. The presynaptic neurons emitted no spontaneous spikes. We considered STDP stimulations with a time difference of $-80 \leq \Delta t \leq 80$ ms and pre-only stimulations when the E1 population was in each state (global up, local up, global down, or local down state). In the case of the pre-post stimulation (i.e., Δt is positive), the stimulation evoked synchronous presynaptic spikes first followed by Δt, a postsynaptic spike. Stimulation was applied if the target state of the E1 population continued to be more than 200 ms, and the interval from the last stimulation was 500 ms or more at the candidate time of the presynaptic stimulation. In the case of the post-pre-stimulations (i.e., Δt is negative), a postsynaptic spike was evoked Δt before the synchronous presynaptic spikes. Stimulation was applied if the above condition was satisfied at the candidate time of the postsynaptic stimulation. The pre-only stimulation was the same as the pre-post stimulation, except that the postsynaptic stimulation was absent. The number of stimulations was fixed at ten. To exclude rare network configurations due to random synaptic connections in which the target states hardly appear, the simulation time was fixed to 50000 ms, and the simulations in which the number of stimulations did not reach ten by the end of the simulation time were excluded from the analysis.

Task simulation. Fig. 5 described the task simulation. The model connection structure was similar to that shown in Fig. 4, containing $N^{\mathrm{ext}} = 80$ presynaptic neurons that had plastic synaptic weights with an initial value of $w_j^{\mathrm{ext}} = 0.09$ mV (Fig. 5*A*). The presynaptic neurons were divided into two populations, G and L, consisting of 40 presynaptic neurons each.

First, we introduced the awake period. While awake, the adaptation current was set to be constant at $I_i^a(t) = 0.025$ mV/ms, in which the neurons showed a continuing depolarized membrane potential similar to the awake firing patterns experimentally observed. In the absence of task-related stimulation, the mean excitatory firing rate during wakefulness was approximately 6.0 Hz. We referred to this firing rate as the baseline firing rate.

Next, we considered a task inspired by the brain-machine-interface task (6) while awake. We assumed that the presynaptic neurons in both G and L populations emit synchronous Poisson spikes at 5 Hz during the task. Task performance was defined as the difference between the mean firing rate of the postsynaptic neuron during the task period and the baseline firing rate. In this setting, the feedforward synaptic weights contributed to the task performance.

Finally, we considered the synaptic changes and improvements in task performance during the post-learning NREM sleep period. During NREM sleep, the dynamics of the adaptation current followed the slow-wave model in the previous section, and the presynaptic neurons emitted synchronous Poisson spikes as memory reactivation at linearly decreasing rates from 7.5 Hz at the beginning of NREM sleep to 5 Hz at the end of NREM sleep. In the following, we compare the results without (Fig. 5) and with (Fig. S7) memory reactivation in the down states. In Fig. 5, the presynaptic neurons in the G and L populations emitted spikes only during the global and local up states of the E1 population, respectively (Fig. 5*B*). In Fig. S7, the presynaptic neurons in the G and L populations emitted spikes during both the up and down states of global and local slow waves surrounding the E1 population, respectively (Fig. S7*B*). In these figures, we extend the time intervals for blocking synaptic plasticity by including the 50 ms margins after. The reason for doing this is that, while synaptic changes due to the synaptic cost term decrease synaptic weight immediately after each presynaptic spike, synaptic changes due to the information term depend on the recent history of neural activity. This difference does not affect model predictions if the input statistics is time-invariant but induces special effects around state transitions due to the temporally unbalance of the

two optimization terms. Note that this difference is an arbitrary model assumption because we can always include some time delays for cost-term-induced changes as well. To exclude this transition effect, we blocked synaptic plasticity, including the 50 ms margins after state transitions.

We define the task neuron's reactivation strength in each state as the increment of firing rate by the task cue reactivation with respect to the baseline firing rate without task cues. Thus, the task neuron reactivation strength $A_Z(t)$ at the time t in global up ($Z = \mathrm{GU}$) and local up ($Z = \mathrm{LU}$) states were defined as

$$A_Z(t) = \frac{\int_0^{T_{\mathrm{conv}}} S_1^E(t-t')Z(t-t')dt'}{\int_0^{T_{\mathrm{conv}}} Z(t-t')dt'} - r_{\mathrm{base}}^Z,$$

with $T_{\mathrm{conv}} = 200000$ ms, $Z(t)$ taking value 1 when the state at the time t is Z and 0 otherwise, and r_{base}^Z representing the baseline firing rate of the state Z. $r_{\mathrm{base}}^{\mathrm{GU}}$ and $r_{\mathrm{base}}^{\mathrm{LU}}$ were set to 5.93 Hz and 7.97 Hz, respectively, according to the mean firing rates shown in Fig. 3F. The task performance was quantified by the above measure using the synaptic weights before and after NREM sleep.

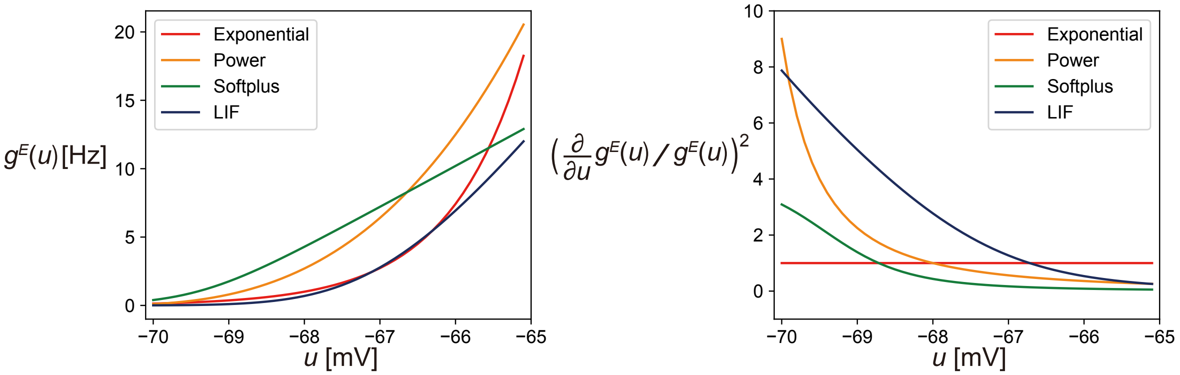

Fig. S1. Synaptic changes in various activation functions. The shape of each activation function (left) and synaptic changes depending on baseline membrane potential in each activation function (right). Four representative activation functions are shown: the softplus function $g^{\mathrm{E}}(u) = r_0^{\mathrm{E}} \log(1 + \exp((u - u_0^{\mathrm{E}})/\Delta u^{\mathrm{E}}))$ with $r_0^{\mathrm{E}} = 1.5$ Hz, $u_0^{\mathrm{E}} = -69.4$ mV, and $\Delta u^{\mathrm{E}} = 0.5$ mV, the power function $g^E(u) = k_p[u - u_{\theta_p}]_+^a$ with $k_p = 0.1$ Hz, $u_{\theta_p} = -71.0$ mV, and $a = 3$, the response function of noisy leaky integrate-and-fire neurons $g^E(u) = \left(\tau_m^E \sqrt{\pi} \int_{\frac{u_{\theta_0} - u}{\sigma}}^{\frac{u_{\theta_1} - u}{\sigma}} \exp(x^2)[1 + \mathrm{erf}(x)]dx\right)^{-1}$ with $u_{\theta_0} = -70.0$ mV, $u_{\theta_1} = -64.0$ mV, and $\sigma = 2.0$ mV, and the exponential activation function $g^E(u) = k_e \cdot \exp(u - u_{\theta_e})$ with $k_e = 0.05$ Hz and $u_{\theta_e} = -71.0$ mV. The synaptic changes (right) were monotonically decreasing in increasing baseline membrane potential except for the exponential activation function, which supports the bias toward synaptic depression in up states with various types of activation functions experimentally observed (see *SI Appendix Methods* for detail).

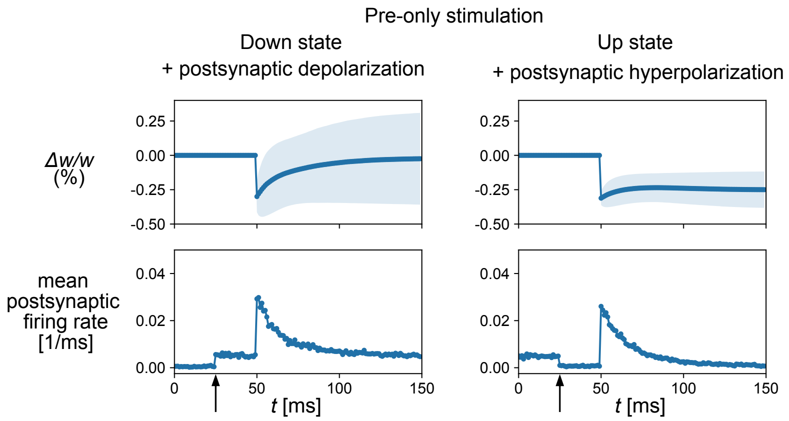

Fig. S2. The mean traces of a synaptic weight and the mean postsynaptic firing rate in the pre-only stimulations, where the stimulated presynaptic neurons emitted synchronous spikes upon the external stimulation at $t = 50$ ms. Artificial postsynaptic depolarization to the up state level in down states and hyperpolarization to the down state level in up states were induced at $t = 25$ ms, as marked by arrows. Note that these artificial manipulations affect the firing intensity $g^{\mathrm{E}}(u)$, but not the mean firing intensity $\overline{g}$, since we assume that the mean firing intensity would be computed from the local population activity in a biological circuit (see Methods and Discussion for details). The synaptic changes were not appreciably different from those in Fig. 2*A* since the effects of the EPSPs on the postsynaptic firing rate dominated those of artificial depolarization and hyperpolarization.

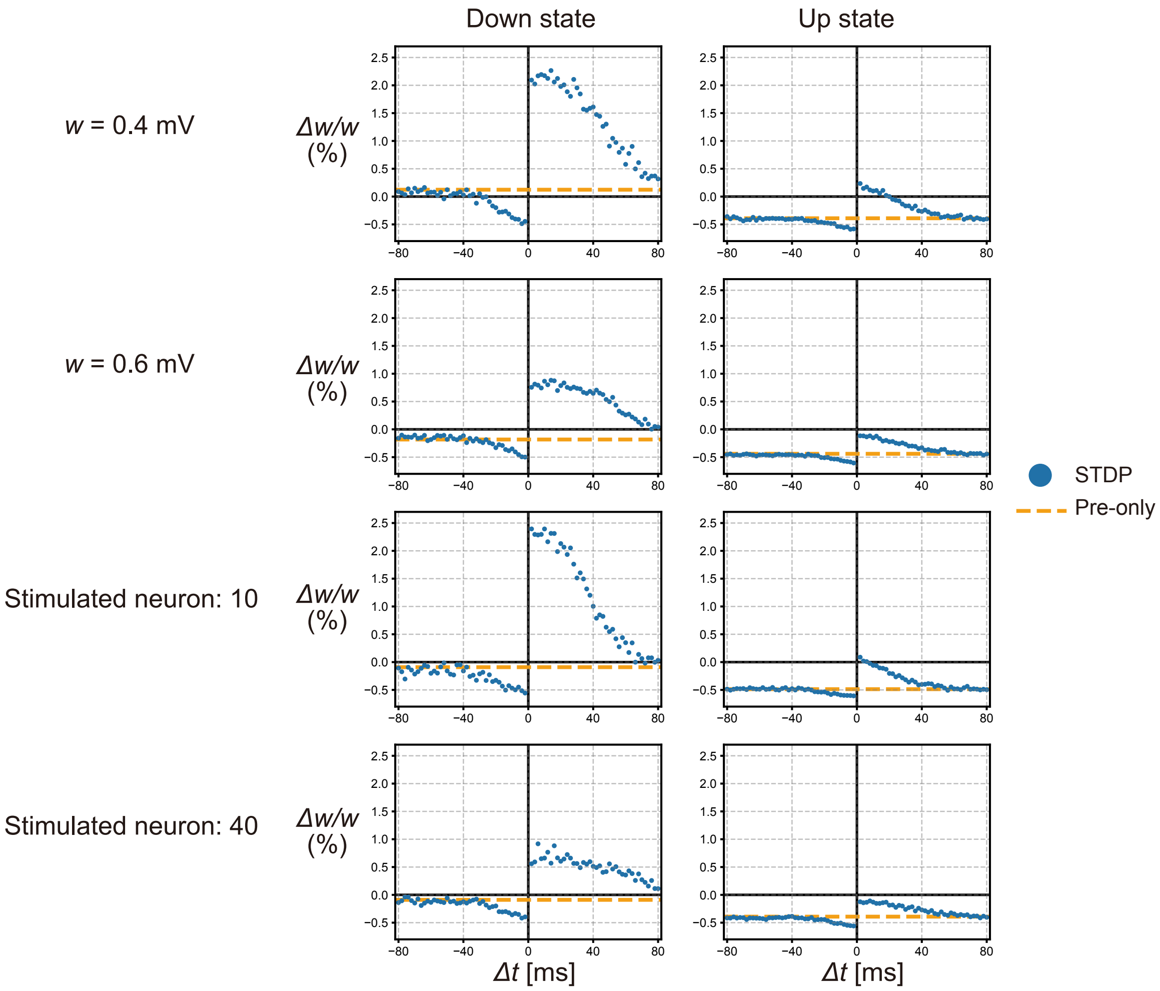

Fig. S3. STDP curves for distinct initial synaptic weights and numbers of stimulated neurons in the single-neuron model.

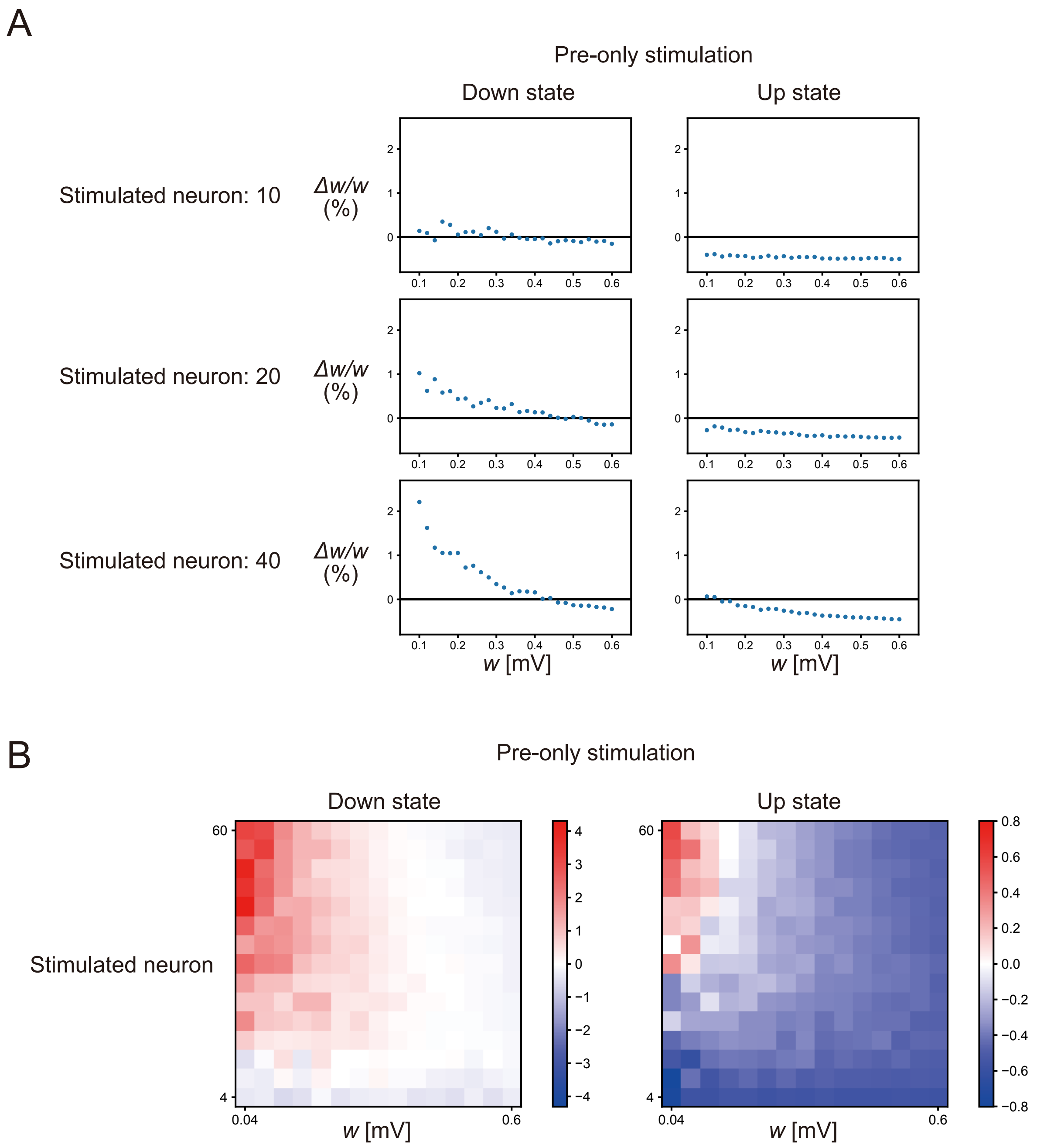

Fig. S4. The synaptic changes by the pre-only stimulations depend on initial synaptic weights and the number of stimulated neurons. (*A*) The synaptic weight changes by the pre-only stimulations were biased toward depression in up states. They also depended on initial synaptic weights and the number of stimulated neurons. (*B*) The synaptic weight changes by the pre-only stimulations. The smaller initial synaptic weights and a larger number of stimulated neurons biased the changes toward potentiation.

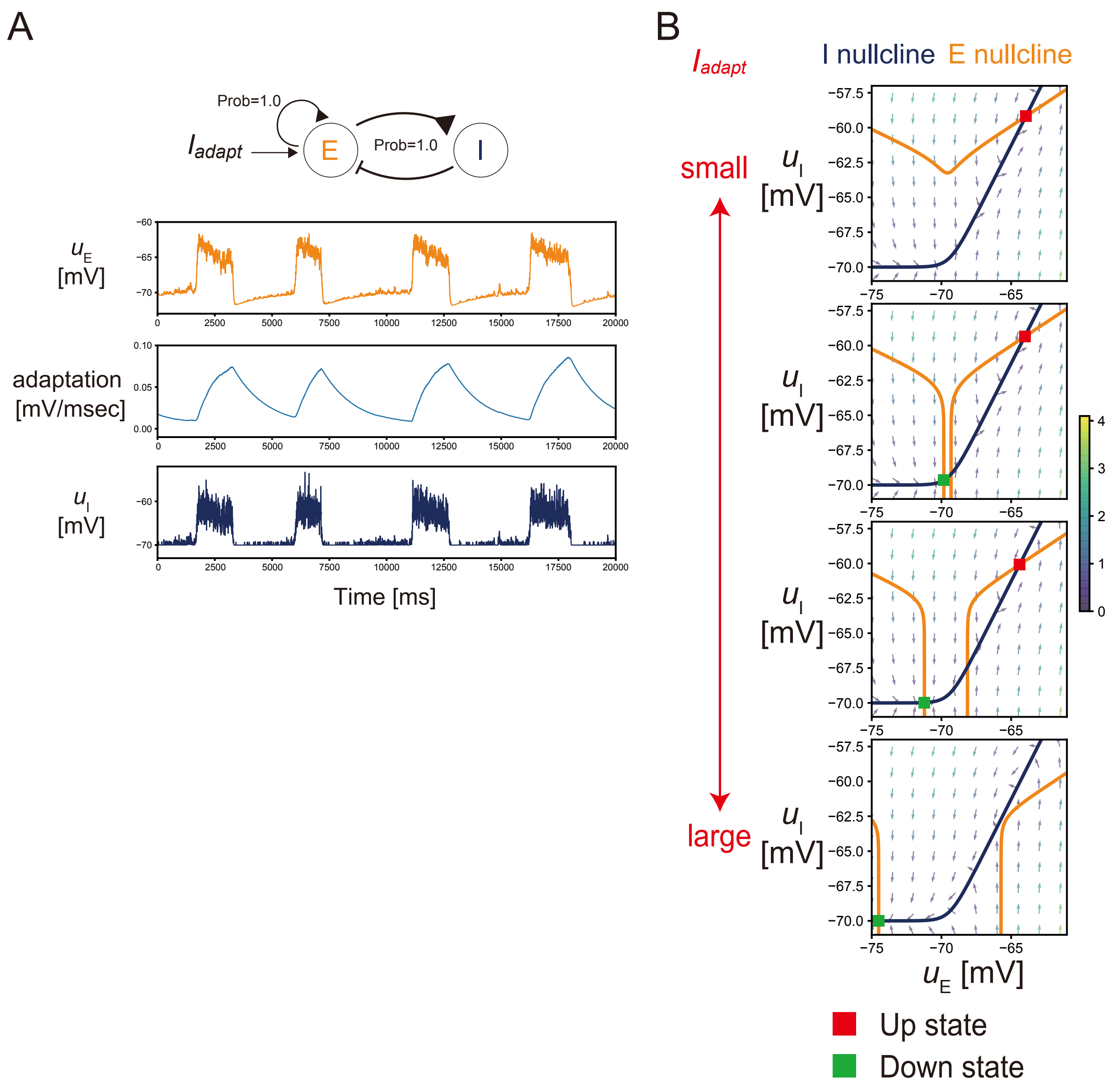

Fig. S5. The spatially homogeneous model with 200 excitatory and 50 inhibitory neurons. (*A*) The membrane potential of excitatory and inhibitory neurons transit between up and down states with changing adaptation currents. (*B*) The phase plane plot of the model. The excitatory nullcline changed with distinct adaptation currents.

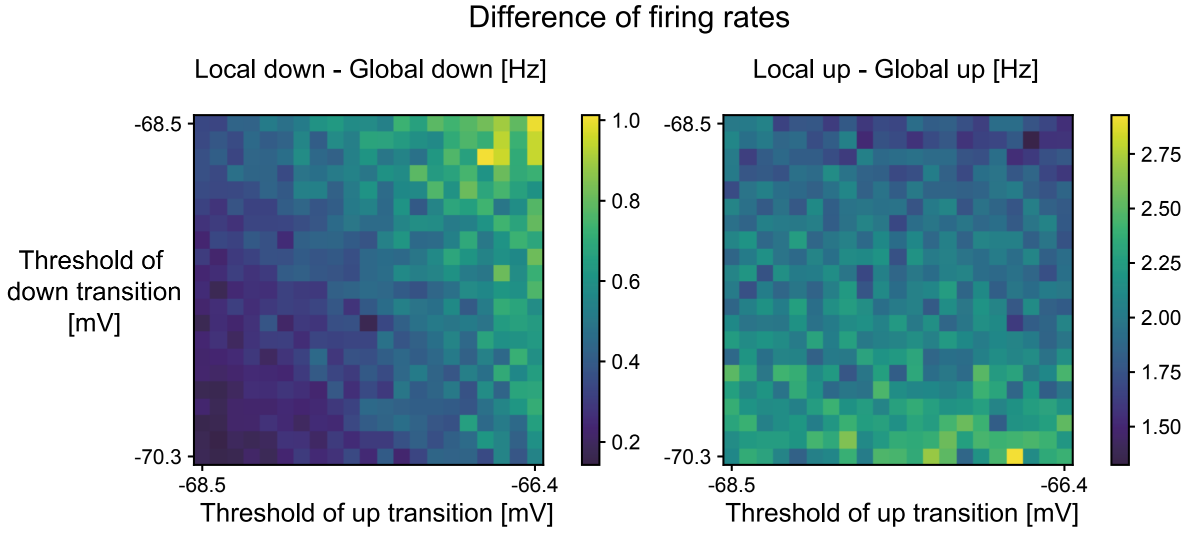

Fig. S6. Changing the threshold of up and down transitions. The firing rate difference of global and local down (left) and up (right) states when the thresholds defining up and down transitions are varied. The threshold of down and up transitions was changed between the peak and bottom of the membrane potential distribution shown in Fig. 3*E*. The firing rate was robustly higher with local than global slow waves in both up and down states.

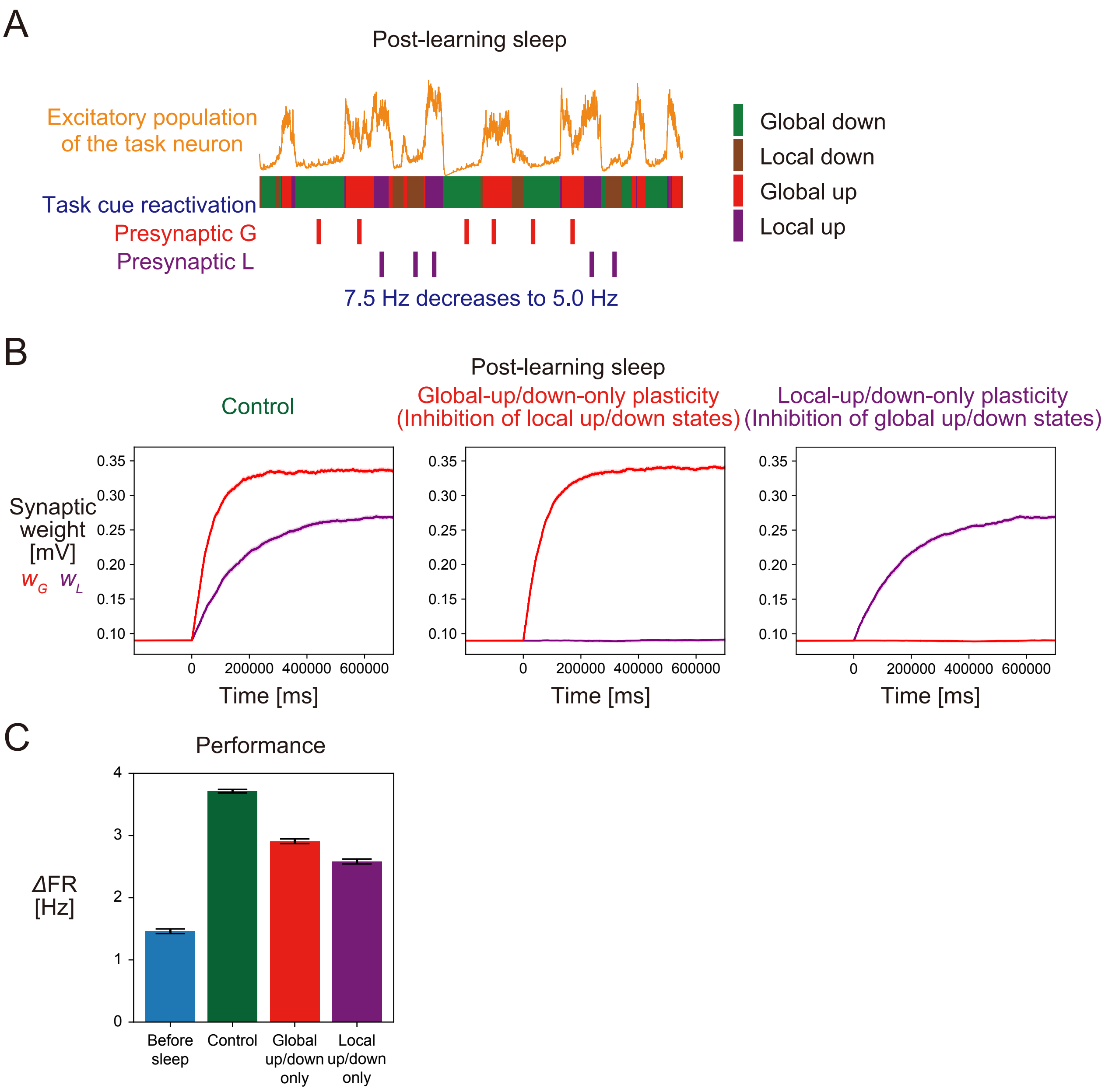

Fig. S7. Changes in synaptic weights and task performance during post-learning NREM sleep in the case that neuronal reactivation during down states also happened. (*A*) A typical mean membrane potential of the E1 population. As neuronal reactivation, the G and L population emitted synchronous spikes during both the up and down states of global and local slow waves, respectively, during the post-learning NREM sleep. The rate of memory reactivation of the task cue was assumed to linearly decrease in time from 7.5 Hz at the beginning to 5.0 Hz at the end of NREM sleep. (*B*) The synaptic changes during the post-learning sleep. The synaptic changes of the G and L populations are shown in red and purple, respectively. Greater synaptic potentiation than Fig. 5 was induced due to the effect of the reactivation during down states. Obviously, no synaptic changes of the population G were observed when its synaptic plasticity was blocked altogether, and so did those of the population L when its reactivation was blocked. The lines and shadows represent the means and SEMs in the 100 trials, respectively. (*C*) The comparison of task performance before and after synaptic changes during sleep. Error bars represent SEM.

References

1. W Gerstner, WM Kistler, *Spiking Neuron Models.* (Cambridge University Press), (2002).
2. R Jolivet, A Rauch, HR Lüscher, W Gerstner, Predicting spike timing of neocortical pyramidal neurons by simple threshold models. *J. Comput. Neurosci.* **21**, 35–49 (2006).
3. NJ Priebe, F Mechler, M Carandini, D Ferster, The contribution of spike threshold to the dichotomy of cortical simple and complex cells. *Nat. Neurosci.* **7**, 1113–1122 (2004).
4. J Anderson, I Lampl, I Reichova, M Carandini, D Ferster, Stimulus dependence of two-state fluctuations of membrane potential in cat visual cortex. *Nat. Neurosci.* **3**, 617–621 (2000).
5. JS Anderson, I Lampl, DC Gillespie, D Ferster, The contribution of noise to contrast invariance of orientation tuning in cat visual cortex. *Science* **290**, 1968–1972 (2000).
6. J Kim, T Gulati, K Ganguly, Competing Roles of Slow Oscillations and Delta Waves in Memory Consolidation versus Forgetting. *Cell* **179**, 514–526.e13 (2019).
